# Unlocking the Essence of Lignin: High‐Performance Adhesives That Bond via Thiol‐Catechol Connectivities and Debond on Electrochemical Command

**DOI:** 10.1002/adma.202510463

**Published:** 2025-08-15

**Authors:** Keven Walter, Dominik P. Hoch, Leon Hertweck, Kannan Balasubramanian, Jonas Geisler, Mathias Röllig, Tilmann J. Neubert, Hans G. Börner

**Affiliations:** ^1^ Humboldt‐Universität zu Berlin Institute of Chemistry Unter den Linden 6 10117 Berlin Germany; ^2^ Humboldt‐Universität zu Berlin School of Analytical Sciences Adlershof Unter den Linden 6 10117 Berlin Germany; ^3^ Bundesanstalt für Materialforschung und –prüfung (BAM) Fachbereich 8.3 ‐ Thermografische Verfahren Richard‐Willstätter‐Straße 11 12489 Berlin Germany; ^4^ Friedrich‐Schiller‐Universität Jena Institute for Technical and Environmental Chemistry Philosophenweg 7a 07743 Jena Germany

**Keywords:** debonding on demand, dopa mimics for microelectronic devices, mussel inspired glue, recycling and circular economy, smart two component (2K) adhesives

## Abstract

The next generation of adhesives requires effective debonding capabilities that can be triggered on demand to enable advanced circular repair and recycling strategies. A new class of lignin‐inspired, two‐component (2K) structural adhesives offers bonding strengths of up to 20 MPa and clean, on‐command electrochemical debonding within 5–30 min. The debonding is induced by a distinct electrochemical oxidation of thiol‐catechol connectivities (TCCs) within the entire adhesive network, enforcing rapid and clean adhesive failure on the cathodic substrate side. The TCC‐functionalities are formed during curing by a thiol‐quinone *Michael*‐type polyaddition, reacting polyester‐based trithiols with tris‐quinones as lignin‐inspired minimal building blocks. The structural adhesive can be fine‐tuned by adjusting the formulation. The addition of carbon black and ionic liquids facilitates the desired electrochemical transformation of TCC‐catechols to TCC‐quinones. Applying only 9 V for 5–30 min, leads to clean debonding with 72–86% loss of shear strength. A comprehensive study of curing, bonding, and debonding behavior by rheological, spectroscopic, and electrochemical investigations reveals the debonding mechanism by correlating catechol oxidation to adhesive performance. The electrochemical debonding capability of TCC‐structural adhesives is demonstrated in a functional prototype, where on‐command detachment of a cover glass from a display device is achieved within 6.5 min.

## Graphical abstract

A novel lignin‐inspired adhesive is presented, that combines the bonding strength of a structural adhesive with on‐demand electrochemical debonding, enabled by biomimetic thiol‐catechol connectivities. Voltage application of only 9 V facilitates electrochemical oxidation of catechols to quinones leading to guided detachment on command. This adhesive significantly improves repair and recycling strategies of e.g. microelectronic devices, which is demonstrated on a display prototype.

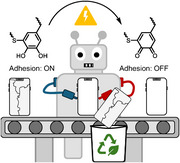

## 1 Introduction

The transformation of chemical reactions, materials, and feedstocks to enhance sustainability poses a significant challenge, yet it also offers tremendous opportunities, e.g., for polymer materials sciences to accelerate the transition to the era of a circular economy.^[^ ^1^ ^]^ Particularly, adhesives are considered to be a class of materials that will benefit from the transition to renewable feedstocks.^[^ ^2^ ^]^ This can be achieved not only by replacing existing fossil‐based products with renewable materials, but also by expanding the scope of functions due to the multifunctional nature of bio‐based building blocks.^[^ ^3^ ^]^ Besides other intriguing functions, like material‐specific adhesion, universal adhesion by adaptive adhesive interfaces, modulus matching by gradual cohesion control, or intrinsic surface drying mechanisms, the capability to trigger debonding can contribute greatly to enabling advanced recycling and repair strategies.^[^ ^4^ ^]^

The design of bioinspired materials fosters enhanced functional diversity, thereby facilitating the development of novel adhesive concepts beyond the common variants. For example, Wilker et al. stimulated the renaissance of protein‐based adhesives by modulating corn‐proteins with the polyphenol tannic acid to achieve degradable, high‐performance glues.^[^ ^5^ ^]^ Messersmith et al. transformed the α‐lipoic acid to a recyclable surgical superglue.^[^ ^6^ ^]^ Moreover, Börner et al. abstracted the thiol‐catechol connectivity (TCC) from the biogenic cysteinyldopa protein crosslink,^[^ ^7^ ^]^ which has been made accessible by a generic and scalable thiol‐quinone *Michael* polyaddition.^[^ ^4^, ^7^, ^8^ ^]^ The incorporation of TCC units into polystyrene (PS) at levels as low as 3 mol% resulted already in an interesting adhesive that overcame the drawbacks inherent to pure PS.^[^ ^9^ ^]^ Additionally, TCC functionalities in a didopa‐based pressure‐sensitive adhesive (PSA) have been demonstrated to outperform fossil TCC‐analogues by debonding with 99% loss of adhesive strength upon chemical oxidation of TCC‐catechols to TCC‐quinones.^[^ ^10^ ^]^

Many examples of catechol‐based adhesives perform on the softer side of the adhesive spectrum, representing hydrogels or pressure‐sensitive adhesives rather than structural adhesives.^[^ ^11^ ^]^ However, recently, structural adhesives that can compete with epoxy glues have been reported more frequently.^[^ ^5^, ^12^ ^]^ These also contain polyphenolic entities from regrowing resources as dopa‐alternatives that pave the way to a new generation of mussel‐inspired adhesives by relying on highly abundant compounds such as lignin or tannins available from biomass or paper‐pulp waste streams.^[^ ^12^, ^13^ ^]^ For instance, a two‐component (2K) TCC‐adhesive has been described, where the guaiacol‐structure elements (G‐units) of lignin have been demethylated and oxidized to *ortho*‐quinones, reacting cleanly with higher molecular weight multithiols (1300 g⋅mol^−1^).^[^ ^12a^ ^]^ In this context, a 2K system refers to a solvent‐free formulation composed of two separate components that, upon mixing, undergo a chemical reaction to form a solid adhesive network.^[^ ^14^ ^]^ The *Michael*‐type polyaddition leads to TCC‐adhesives with 40–55 wt.% lignin content, reaching up to 13 MPa adhesive shear strength, showing no acute toxicity and efficiently gluing living corals under seawater.

Strong and resilient structural adhesives are essential for modern production lines, but pose challenges at end‐of‐life, which are particularly critical for recycling or repair strategies of microelectronic devices.^[^ ^4^, ^15^ ^]^ Therefore, debonding methods for such adhesives are currently being developed, which demand the design of trigger‐response couples. Triggers like heat, electric power, light, or chemical agents are used to induce an appropriate chemical or physical response in the polymer that weakens the adhesive bonding.^[^ ^4g–j^ ^]^ TCC‐adhesives utilize the potency, performance, and robustness of catechols as the key interactive sites. They offer inherently an intriguing debonding mechanism by chemical/electrochemical oxidation (trigger) of catechols to quinones (response).^[^ ^10^, ^16^ ^]^ Messersmith et al. proved ≈80% difference in adhesive strength between catechol and quinone functionalities with single molecule force experiments.^[^ ^16a^ ^]^ The concept was translated to an underwater application by Lee et al. using electrochemical modulation of the pH to shift the catechol‐quinone equilibrium.^[^ ^16b^ ^]^ For TCC‐adhesives, the debonding‐on‐demand has been demonstrated with chemical oxidants as triggers.^[^ ^10^ ^]^ Although the use of a chemical oxidant has the advantage of providing triggers with appropriate reactivity at defined times, the rigid adhesive interface of structural adhesives might impede the accessibility of the trigger. The electrochemical transformation of, e.g., didopa catechols to bisquinones occurs in a rather controlled manner,^[^ ^17^ ^]^ making this trigger highly interesting for adhesives used in microelectronic devices or the automotive sector. Typically, electrically and electrochemically debondable structural adhesives are subject to more extreme conditions for deactivation, which may result in complex decomposition reactions.^[^ ^18^ ^]^ In this context, the realization of defined electrochemical reactions in structural adhesives remains challenging, but studies on soft, catechol‐containing adhesives have clearly demonstrated that the catechol–quinone redox equilibrium can be effectively exploited for debonding applications.^[^ ^10^, ^16^, ^19^ ^]^ Existing strategies, including chemical triggering with oxidizing agents such as sodium periodate and electrochemical approaches relying on water electrolysis to shift catechols to quinones via local pH modulation, have shown potency. Nevertheless, their reliance on aqueous environments imposes constraints for the microelectronic sector, and highlights opportunities to develop alternative, non‐aqueous systems.

**Figure 1:**
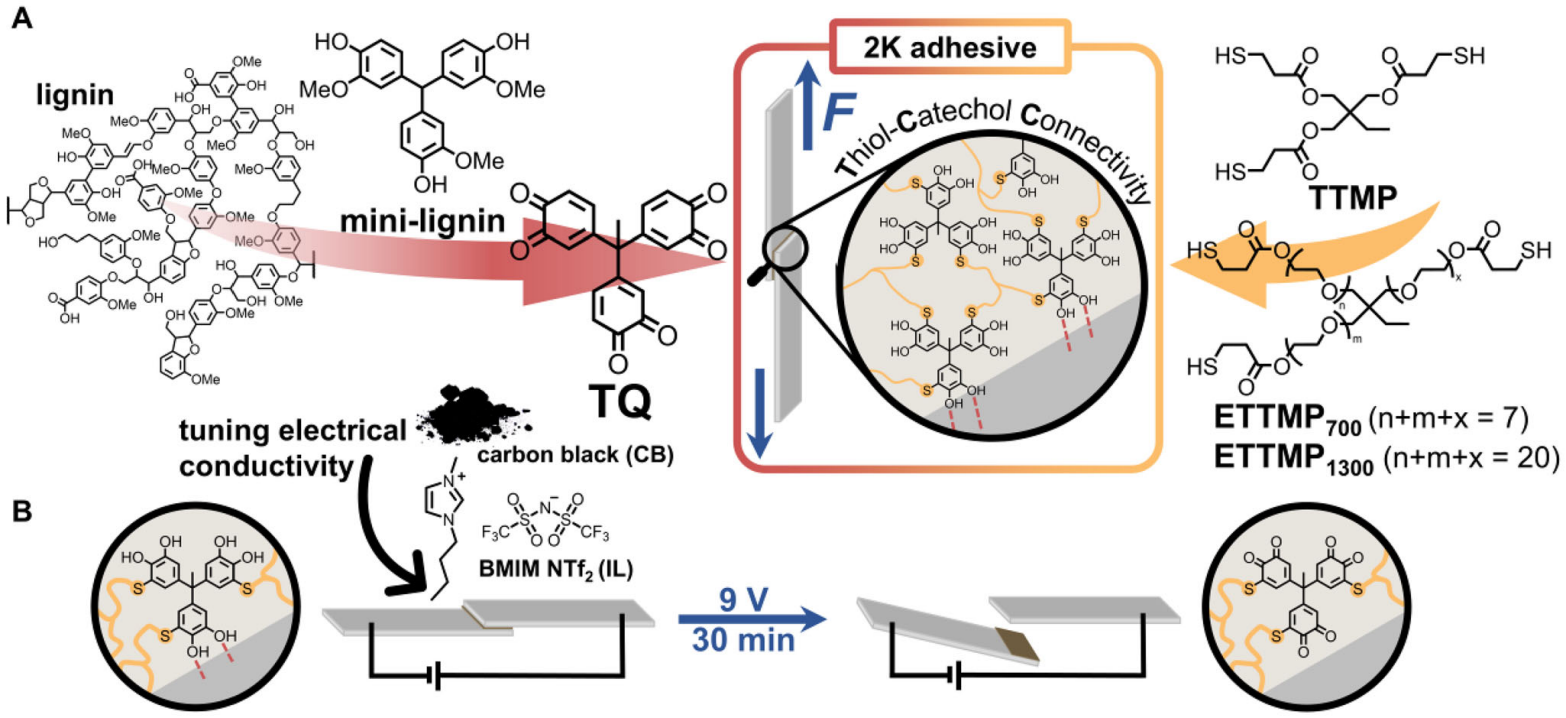
A) Molecular design of the two‐component (2K) adhesive: Trimeric *ortho‐*quinones (TQ), inspired by lignin motifs, react with multithiol crosslinkers to form thiol‐catechol connectivity (TCC) networks. B) Concept of electrochemical debonding, where the applied voltage oxidizes TCCs to generate *ortho‐*quinones, enabling specimens to debond and detach.

Here, we present a bioinspired strategy, in which small lignin‐inspired building blocks are adapted to TCC‐chemistry to enable the reaction with lower molecular weight multithiols, forming strong TCC‐adhesive networks, in which the potent catecholic adhesion motifs are retained and available for debonding applications (**Figure** **1**). The resulting 2K adhesives achieve high shear strengths on aluminum. Upon the incorporation of conductive additives, they facilitate debonding through the electrochemical oxidation of TCC‐catechols, which significantly decreases the shear strength of the adhesive on command.

## 2 Results and Discussion

While a previously introduced lignin‐TCC adhesive cures into tough, rubbery materials that seem to be ideal for anchoring corals in the turbulent seawater environment,^[^ ^12a^ ^]^ the densification of the material network promises improved adhesive properties for dry conditions. Lower molecular weight multithiols such as 1,1,1‐tris(hydroxymethyl)ethane tris(3‐mercaptopropionate) (TTMP, 398 g⋅mol^−1^) appear less compatible with activated lignin of 3–6 kg⋅mol^−1^, as they fail to establish a sufficiently robust cohesive network by bridging multiple lignin molecules as net‐points, likely due to steric constrains and/or limited miscibility with the lignin phase.^[^ ^5^, ^12^ ^]^ Consequently, *mini‐*lignin building blocks in the range of 300–400 g⋅mol^−1^ promise to yield improved properties.^[^ ^20^ ^]^ Unlike the lignin‐based adhesive, which exhibited a lower density of TCC–catechol functionalities, a synthetically defined, low‐molecular‐weight mini‐lignin introduces a higher density of redox‐active TCC sites within a uniformly crosslinked thermoset matrix. This feature appears to be advantageous for oxidative switching, facilitating efficient debonding. Trimeric G‐units can be obtained by *Friedel‐Crafts* acylation of guaiacol and vanillin, which both can be sourced from lignin itself.^[^ ^21^ ^]^ These *mini‐*lignins have previously been used to introduce, for instance, epoxy entities for the synthesis of adhesives.^[^ ^21^, ^22^ ^]^ However, the underlying polymer chemistry relied on established lignin modification strategies that usually consume the valuable phenolic functionalities, thereby reducing relevant interaction motifs.^[^ ^12^, ^23^ ^]^

4,4′,4″‐methanetriyltris(2‐methoxyphenol) (*mini‐*lignin, Figure 1A) was accessible in 100 g reaction scale by adapting procedures of Zhang and coworkers.^[^ ^21a^ ^]^ However, neither the direct oxidative *O*‐demethylation reaction by iodoxybenzoic acid (IBX),^[^ ^24^ ^]^ nor a two‐step procedure performing selective *O*‐demethylation with boron tribromide^[^ ^25^ ^]^ and subsequent oxidation by various reagents such as IBX, sodium periodate, or ceric ammonium nitrate proved to be successful (Section S4.1,S4.2 in Supporting Information). Although the intermediate 4,4′,4″‐methanetriyltris(catechol) could be isolated (Section S3.2, Supporting Information), the desired *mini‐*lignin trisquinone had a pronounced tendency for oxidative coupling, as all attempts to isolate it resulted in insoluble products with an intensely dark color. Considering structural similarities to triphenylmethane dyes, the abstraction of the central tritylic proton gives rise to a fully conjugated π‐system that might render oxidative coupling feasible.^[^ ^26^ ^]^ Similar side reactions have been observed for related structures derived from bisphenol (BP) derivatives with α‐CH‐substituents, preventing the isolation of bisquinones from, e.g., BPE or BPF.^[^ ^8b^ ^]^

**Figure 2:**
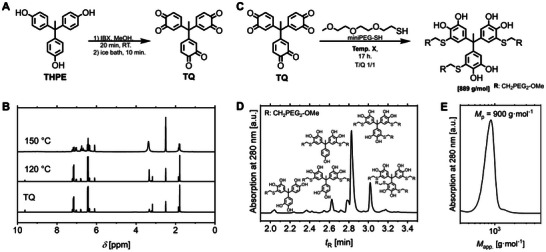
A) Synthesis, B) stability testing, and C) reactivity of TQ. Thermal stress experiments of dry powder TQ in air at different temperatures for 48 h with subsequent ^1^H NMR analysis of samples in DMSO‐*d*_6_ (B), and model bulk reaction at 120 °C of TQ with *mini*‐PEG‐SH (C), and the analysis of the reaction mixture by D) UHPLC and E) THF‐GPC.

After the identification of the central proton as the point of concern, the introduction of a central alkyl substituent should suppress side reactions and allow the isolation of a stable trisquinonic monomer. A single step reaction enabled conversion of 1,1′,1″‐tris(4‐hydroxyphenyl) ethane (THPE) into the corresponding trisquinone (TQ; 4,4′,4″‐(ethane‐1,1,1‐triyl)tris(3,4*‐ortho*‐benzoquinone)) by a metal‐free IBX‐mediated oxidation (Section S3.4, Supporting Information). Adapting the previously reported oxidation route in methanol at room temperature with 1.5‐equivalents excess of IBX per phenolic entity,^[^ ^8a^ ^]^ quantitative quinone formation occurred rapidly, and the red TQ precipitated out of solution. The separation of TQ from the remaining IBX excess was hampered due to their similar solubilities, preventing a practical workup procedure. However, full reduction of TQ to catechol species, chromatographic separation and re‐oxidation provides TQ with 99+% conversion of phenol groups (Figure S4, Supporting Information). The successful conversion of THPE to TQ was confirmed by ^1^H NMR, Fourier‐transform infrared spectroscopy (FTIR), and ultra‐high‐performance liquid chromatography (UHPLC). The reaction conditions were optimized to improve scalability and compound economy. By reducing the IBX excess to 0.98 eq. per phenol the oxidation reaction of THPE fully consumed IBX, and the precipitated TQ was isolated by filtration and washing, to give the desired product in 75% isolated yields (**Figure** **2**A). The use of IBX in a marginal sub‐stoichiometric manner permitted oxidation of phenol residues to quinones with 94% conversion as quantified by ^1^H NMR (Figure S5, Supporting Information). In addition to the TQ, only bisquinone derivatives and no mono‐oxidation product were found. It is crucial to note, that such a product distribution will not hamper the anticipated 2K reaction of multifunctional components, as the polyaddition reaction will not be terminated.

In order to elucidate the possible curing temperature window, the thermal stability of the TQ was investigated with respect to potential unwanted thermally induced side reactions.^[^ ^27^ ^]^ Thermogravimetric analysis (TGA) under an argon atmosphere revealed no visible degradation to occur below 190 °C, with a 5% degradation temperature (*T*_5%_) of 330 °C (Figure S12, Supporting Information). Subsequently, a thermal stability test under air was conducted by stressing TQ samples at different temperatures for 48 h. ^1^H NMR measurements proved structural integrity of TQ until 120 °C, which is often the upper limit of curing temperatures for professional 2K adhesives (Figure 2B). At 150 °C decomposition of TQ takes place before the carbonization was found at 180 °C (Figure S13, Supporting Information).

Prior to the synthesis of crosslinked 2K adhesive resins, the TCC formation along the thiol‐quinone Michael‐type addition mechanism was proven. Consistent with previously reported bisquinones,^[^ ^8b^ ^]^ TQ reacted cleanly with ethanethiol in *N*‐methyl pyrrolidone, and the reaction was completed at room temperature after 15 min as indicated by UV/Vis spectroscopy and UHPLC analyses (Figure S14, Supporting Information). To proceed closer to the viscosity and polarity of the 2K adhesive mixture, a model was used to examine the reaction of TQ with a liquid triethylene glycol monothiol (*mini*‐PEG‐SH) (Section S3.5, Supporting Information). The bulk reaction tests were conducted by mixing TQ with *mini*‐PEG‐SH in a stoichiometric ratio based on functionalities, using a bladeless shear mixer. Subsequent curing was performed at 120 °C for 17 h (Figure 2C). FTIR, UHPLC, and ^1^H NMR analysis suggested the successful conversion of the TQ with *mini*‐PEG‐SH. UHPLC revealed the formation of different *Michael*‐type adducts, with the triple‐addition product having three TCC‐bound *mini*‐PEG‐SH as the main product (Figure 2D). Besides that, also the tetra‐ and double‐addition products could be found with 24% and 10%, respectively. Due to the statistical nature of addition, and given the fact that redox exchange between quinones and TCC catechols can lead to partial reoxidation of the TCCs, a product distribution is to be expected. Previous model studies with low‐molecular‐weight didopa quinone show that TCC formation predominates^[^ ^17^ ^]^ suggesting that these side reactions do not significantly hinder polyaddition or resin formation.^[^ ^28^ ^]^

Interestingly, the curing temperature in bulk reaction could be lowered down to 60 °C, while still achieving full conversion of TQ after 36 h. The FTIR spectra demonstrated the consumption of the quinone species, as evidenced by the disappearance of the quinone vibration band at ν  =  1660 cm^−1^ (Figure S21, Supporting Information). Furthermore, gel permeation chromatography (GPC) excluded the formation of higher molecular weight polymerization products by, e.g., quinone‐catechol crosslinking (Figure 2E).

**Figure 3:**
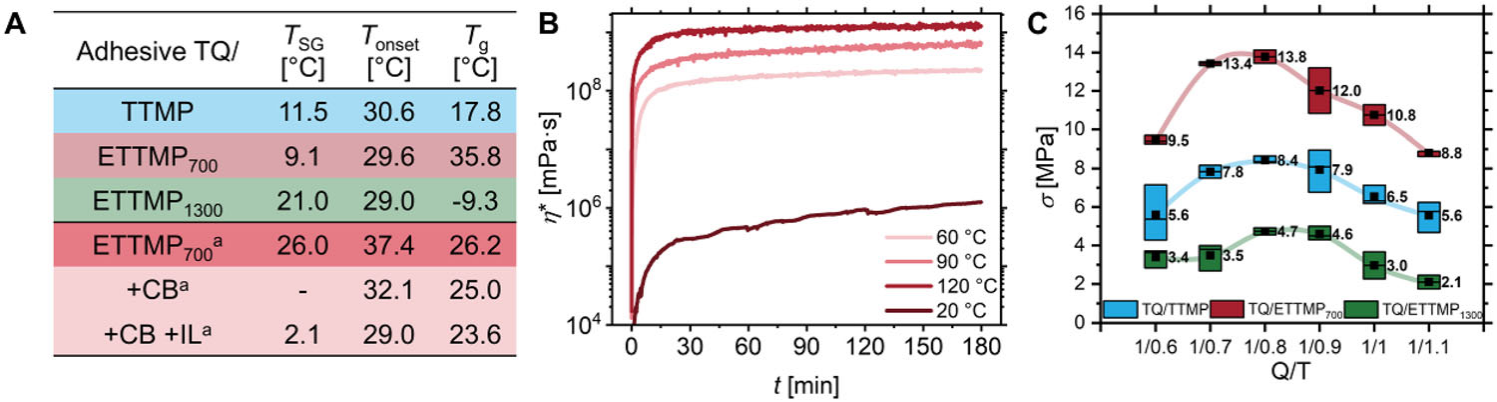
Curing and bonding of various TQ‐based 2K TCC‐adhesives. Sol‐/gel‐transition temperatures (*T*_SG_) determined by rheology and onset temperature (*T*_onset_) for the curing reaction with resulting glass transition temperatures (*T*_g_) determined by DSC (A; (a) Q/T = 1/0.8). Isothermal functions of the complex viscosity (η*) at temperatures of 20 °C, 60 °C, 90 °C and 120 °C exemplarily shown for TQ/ETTMP_700_ (B). Shear strengths (*σ*) of adhesive mixtures on aluminum displayed as a boxplot (square = average value, line = median) with various Q/T ratios cured at 120 °C for 17 h (C).

To realize adhesives with structural integrity and high strength, a bulk 2K resin synthesis was performed. The 2K adhesive was composed by mixing TQ with different liquid three‐arm star polymers, exhibiting poly(ethylene glycol) (PEG)‐based arm segments and three ω‐thiol chain end functionalities (Figure 1). Despite their chemical similarities, the trithiol components differ in viscosities and thiol densities as the molecular weights range from the smallest TTMP, to the ethoxylated trimethylolpropane tri(3‐mercapto‐propionate) (ETTMP_700_) with average *DP*_n,PEG‐arm_ of ∼2, to the largest, ETTMP_1300_ trithiol, having *DP*_n,PEG‐arm_ of ∼7 (Table S2, Supporting Information). The mixing of the 2K systems with a constant quinone/thiol ratio (Q/T) of 1/1, was carried out with the bladeless mixer. The formulation adhered to some principles of green chemistry and was developed to work entirely solvent‐free, as the liquid trithiol components ensure sufficient flowability and facilitate homogeneous mixing with the solid TQ precursor and formulation additives. The curing behavior of different 2K mixtures was investigated via rheological and differential scanning colorimetry (DSC) analysis (**Figure** **3**A). Both methods revealed conclusively that curing occurs upon heating, with sol‐gel transition temperatures (*T*_SG_) in the range of 9–21 °C (rheology, Figure S17, Supporting Information) and on‐set temperatures (*T*_onset_) of ≈30 °C (DSC, Figure S27, Supporting Information). Rheology can detect mechanical changes at a very early stage, whereas DSC does not register significant heat release until the reaction reaches a certain intensity.^[^ ^29^ ^]^ Additionally, rheology clearly showed that an increased curing temperature results in enhanced final strength (cf. TQ/ETTMP_700_ in Figure 3B, and other mixtures in Figure S19, Supporting Information). FTIR spectroscopy provided insight into the resin forming reaction at the molecular level, by witnessing the disappearance of the *ortho*‐quinone vibration band at *ν*  =  1660 cm^−1^ upon heating, while the hydroxy vibrations ≈3300 cm^−1^ reappeared in accordance with the formation of TCC catechols (Figure S22, Supporting Information). Ultimately, the TCCs could be found as dominating molecular crosslinking principle in the resin, by hydrolysis of the 2K resin after curing. The representative TQ/TTMP adhesive was hydrolyzed under acidic conditions to break the ester bonds of the TTMP units. The resulting fragments could be analyzed by UHPLC to prove the formation of TCCs by showing *Michael*‐adducts of TQ with mercaptopropionic acid (Figure S16, Supporting Information).

The cured adhesives demonstrated high thermal stability, as evidenced by TGA analysis, which showed no significant degradation below 300 °C. The *T*_5%_ were found between 305 °C and 332 °C, with maximal degradation rates (*T*_max_) located in the range from 344 °C to 383 °C from the smallest to the largest trithiol used (Figure S27, Supporting Information). To assess the adhesion strength (*σ*), lap shear tests with construction aluminum specimens were performed. Initially, all three 2K systems were tested at stoichiometric Q/T feed composition of 1/1 (Section S4.6, Supporting Information). The glued specimens were cured at 60 °C, 90 °C, or 120 °C for 17 h, and apparently, the curing temperature had a strong effect on adhesive properties. When cured at 120 °C, the base 2K mixtures reached already remarkable adhesive shear strengths, with TQ/ETTMP_700_ providing the optimum and entering with *σ* = 10.8 ± 0.5 MPa the higher performance regime of structural adhesives. The other trithiols TQ/TTMP and TQ/ETTMP_1300_ performed significantly weaker, giving *σ* = 6.5 ± 0.7 MPa and *σ* = 3.0 ± 0.4 MPa, respectively. Lowering the curing temperature to 90 °C reduces dramatically the adhesive performance as TQ/ETTMP_700_ and TQ/TTMP reached only *σ* = 2.4 and 0.5 MPa, respectively, and at 60 °C the gluing fails for these mixtures. It might be reasonable to assume that the adhesive resin network benefits from the last few percent of reaction conversion, which significantly increases strength but obviously requires higher curing temperatures. Interestingly, TQ/ETTMP_1300_ shows the highest shear strengths of *σ* = 3.8 ± 0.3 MPa at 90 °C and brings measurable strength also at 60 °C. The ETTMP_1300_ offers the highest flexibility, promoting the dynamics for TCC network formation at lower temperatures, even though the resulting network has the lowest cross‐link density due to the high molecular weight trithiol being used. Subsequently, for all mixtures, the Q/T stoichiometry was systematically varied in the window between 1/0.6 and 1/1.1 (Figure 3C). Within this range, mixability was fully given, and curing was carried out at temperatures of 120 °C, which proved to be most promising. Shear testing revealed a consistent trend for all different trithiols, thereby highlighting the generic character of the thiol‐quinone *Michael* polyaddition. In all cases, a slight excess of quinones was favorable to reach higher shear strengths, and the optimum was found at a Q/T ratio of 1/0.8. The highest strength of *σ*  =  13.8 ± 0.4 MPa was observed for the TQ/ETTMP_700_ adhesive, which represents a remarkable performance for a 2K base mixture, without any sophisticated optimization through advanced formulations. Considering the 3D‐resin character of the cured adhesives, it is not surprising that cohesion is strong and the sample failure mode was predominantly by adhesive fracture mechanisms (Figure S33, Supporting Information). Nevertheless, a high fraction of TCC functionalities should, in principle, result in robust surface interactions and contribute to cohesion.^[^ ^9^ ^]^

**Figure 4:**
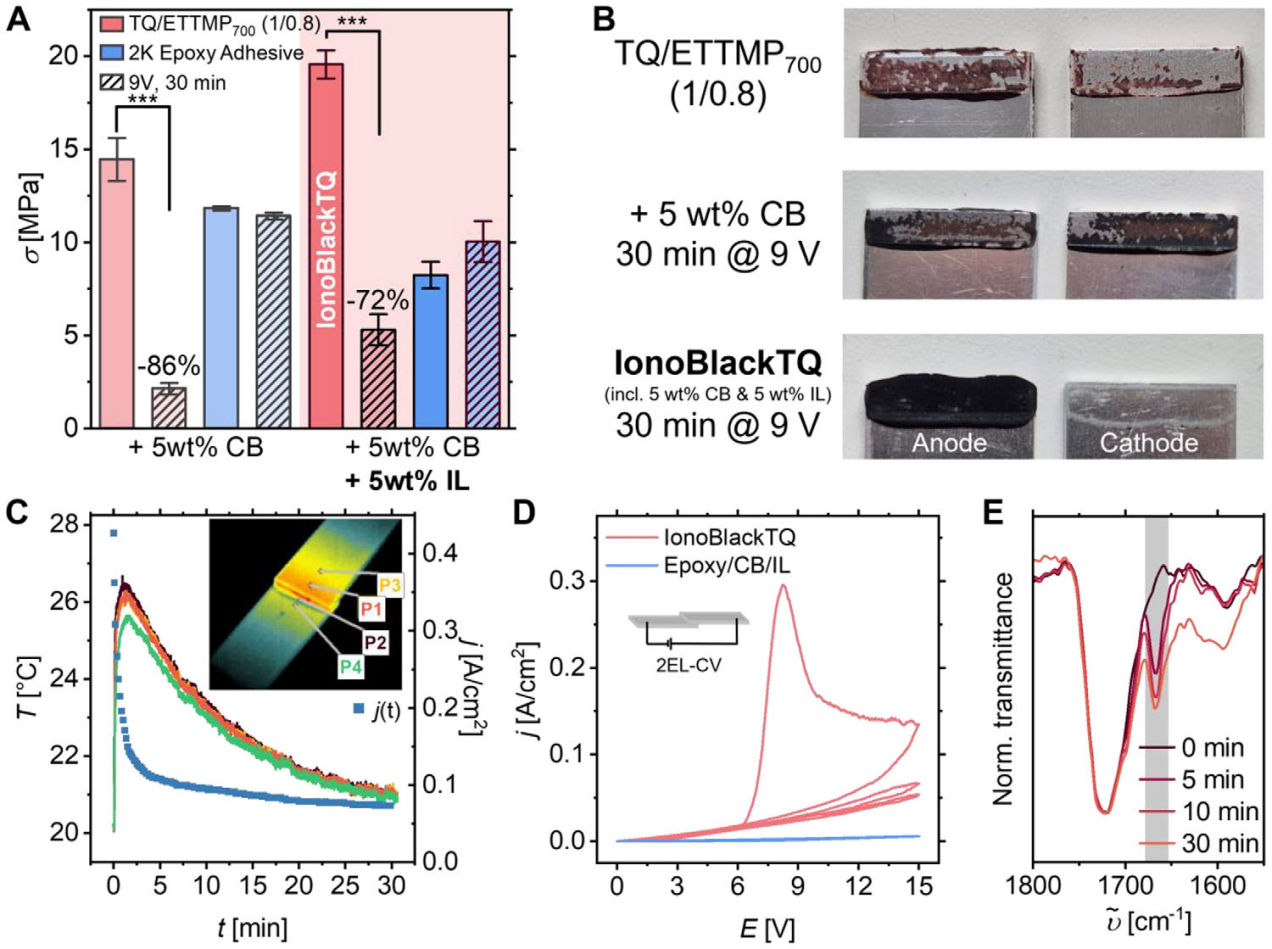
A) Electrochemical debonding of IonoBlackTQ adhesive compared to a commercial epoxy system of similar strength. Shear strength of TQ/ETTMP_700_ (1/0.8) with carbon black (CB, 5 wt.%) as well as with CB (5 wt.%) and BMIM·NTf_2_ as ionic liquid (IL, 5 wt.%) on aluminum before and after voltage application (9 V for 30 min) in comparison to commercial epoxy glue with the same additives. Significance level: *p* < 0.001 (***). B) Fracture pattern of the different formulated TQ/ETTMP_700_ (1/0.8) adhesives on aluminum. C) Temperature tracking via IR camera during the debonding process of TQ/ETTMP_700_/CB/IL at various points (P1 – P4) on the specimens in comparison to the current density (*j)* flowing through the adhesive. D) Two‐electrode cyclic voltammograms (2EL‐CV) of IonoBlackTQ (red) and epoxy/CB/IL (blue) between two aluminum substrates with a scan rate of 10 mV⋅s^−1^. E) FTIR analysis before and after voltage application (9 V for up to 30 min) of TQ/ETTMP_700_/CB/IL (1/1.1), *ortho*‐quinone vibration band at 1664 cm^−1^ is highlighted in grey (normalized to the ester carbonyl band at 1739 cm^−1^).

After optimizing the adhesive system of the TQ‐based adhesive, the concept of electrochemically triggered direct oxidation of TCC‐functionalities was implemented for the structural adhesive TQ/ETTMP_700_. Symmetrical adhesive joints of aluminum substrates bonded with the TQ/ETTMP_700_ adhesive were investigated, allowing for the application of an electrical potential through the metallic substrates. Additionally, conductive additives were required to overcome the insulating nature of the cured 2K base mixture, which does not respond to voltage application on its own (Figure S31, Supporting Information). Intensive iterative optimization resulted in a formulation with remarkable bonding and debonding performance (Figure 1A and **Figure** **4**). The TQ/ETTMP_700_ mixture with a Q/T‐ratio of 1/0.8 was filled with 5 wt.% carbon black (CB) and 5 wt.% of the ionic liquid (IL) 1‐butyl‐3‐methylimidazolium bis(trifluoromethylsulfonyl)imide (BMIM·NTf_2_). The formulated 2K adhesive termed **IonoBlackTQ** exhibited an even enhanced shear strength of 19.6 ± 0.7 MPa, outperforming the base adhesive (without conductive additives) by more than 30%. The improved adhesion is likely due to the contribution of both additives to the adhesive profile. CB filler particles improve cohesion, while the low‐molecular‐weight IL increases wettability and reduces brittleness. Upon applying a constant voltage of 9 V for up to 30 min, the IonoBlackTQ adhesive exhibited a distinct electro‐triggered debonding behavior. Adhesive failure occurred at ≈5 MPa on the cathodic substrate side, corresponding to a 72% reduction in initial shear strength (Figure 4A,B). Scanning electron microscopy (SEM) confirmed a clean adhesive failure, with electrochemical debonding exposing the aluminum substrates down to the machining marks, while largely preserving the microstructural integrity (Figure S41, Supporting Information). This contrasts sharply with the mixed failure modes observed in the absence of electrochemical stimulation. Notably, this electrochemical debonding effect was not limited to aluminum substrates. Other electrically conductive materials, such as steel, coated glass, or carbon‐doped plastics, also proved compatible with IonoBlackTQ adhesives, achieving 42–87% reduction in adhesive strength by electrochemical debonding (Figure S34, Supporting Information). In contrast, solvent‐induced swelling resulted in substantially smaller reductions in adhesive strength. For example, after two hours of immersion in an acetone solvent bath, there was only a ≈30% decrease, while exposure to ethanol, isopropanol, or water resulted in even less pronounced effects (Figure S35, Supporting Information). These results highlight the intrinsic stability of the adhesive resin matrix against chemical softening and emphasize the chemical resistance of IonoBlackTQ adhesive joints as an important performance parameter for demanding applications.

The enhanced shear strength and the debonding capability of the IonoBlackTQ system are intriguing, however, the catechol oxidation as the source of the debonding effect needs to be proven. The first indication for the electrochemical debonding capability of the TCC‐adhesive being linked to the TCC‐catechols was found by testing an analogue formulation, but using a commercially available 2K epoxy adhesive instead of the TCC‐adhesive. The epoxy‐based adhesive filled with only CB or CB/BMIM·NTf_2_ proved to be unaffected by voltage application (Figure 4A). Apparently, the fillers facilitate the electrochemical reactions that trigger detachment, but were not themselves causing detachment. This distinction is significant because ILs are frequently regarded as the sole active species in electrochemically debondable adhesives, taking direct roles by electromigration and/or electrodecomposition.^[^ ^18d,f–i^ ^]^ The IL is required to integrate ionic conductivity into the TQ/ETTMP_700_ adhesive. However, as the sole additive, BMIM·NTf_2_ was also ineffective in establishing electrochemical debonding properties in the TCC‐adhesive. Even at up to 30 wt.% IL feed (Figure S31, Supporting Information) only small amounts of current were passing through the layer having a resistance in the MΩ range. To improve the conductivity of the layer and allow for a faster charge transfer, CB was added to the TCC‐adhesive. In contrast to minor effects of the IL, the incorporation of 5 wt.% CB notably facilitated electrochemical debonding effects and improved the conductivity yielding CB‐filled adhesives with low resistance of 100–500 Ω, which was slightly lower than that of the IonoBlackTQ with typically 0.5‐2 kΩ. The proportion of CB was also critical as too much leads to high electrical conductivity, which prevents an electrochemical reaction from occurring, while too little does not introduce notable conductivity to the layer. Interestingly, the application of 9 V for 30 min on 5 wt.%‐CB‐filled TCC‐adhesive resulted in a reduction of the shear strength from 14.5 ± 1.0 to 2.1 ± 0.2 MPa by 86%, which is in a similar range to the fully formulated IonoBlackTQ (Figure 4A). However, in the case of only CB filler, the fracture patterns indicated a non‐preferred mixed failure mode. This suggests that the added IL acts in synergy with CB to enable the clean debonding pathway in IonoBlackTQ (Figure 4B) as proven by SEM surface morphology analysis (Figure S41, Supporting Information). A notable difference between both formulations was found during the voltage application by comparative thermal tracing of the debonding process in IonoBlackTQ and the CB‐only filled TQ/ETTMP_700_ adhesive using an IR‐camera. The fully formulated adhesive IonoBlackTQ exhibited only marginal increases in temperature above room temperature during voltage application, with peak temperatures of ≈26 °C (Figure 4C). On the contrary, the only CB‐filled control TCC‐adhesive experienced a severe rise in temperatures to 75 °C (Figure S36, Supporting Information). During control experiments, glued aluminum specimens could be heated up to 200 °C for 30 min without a significant decrease in shear strength, allowing for the conclusion that thermally induced debonding effects could be excluded for IonoBlackTQ (Figure S31, Supporting Information).

The application of the debonding voltage to the IonoBlackTQ initially resulted in high currents, which over time decreased substantially (Figure 4C). To confirm the Faradaic origin of this current decay, two‐electrode cyclic voltammetry (CV) experiments of the adhesive films in between the two aluminum substrates were conducted (Figure 4D). Interestingly, the CVs showed the same features for IonoBlackTQ and the TCC adhesive filled with CB only (Figure S38B, Supporting Information). In the first cycle at a scan rate of 10 mV⋅s^−1^, a peak current was reached at ≈8 V with an onset at ≈6 V. The electrochemical transformations were not reversible, as the reverse scan and the subsequent cycles were featureless, implying a completed reaction that supports the debonding process. Upon increasing the scan rate, a systematic increase of the peak current and position was found, which further underlines the Faradaic origin of the current (Figure S38A, Supporting Information). Again, the epoxy‐based analogue adhesive formulation (Epoxy/CB/IL), was utilized as a control and proved the absence of any electrochemical reaction or Faradaic current in the applied voltage range of up to 15 V (Figure 4D). This supports the conclusion that the electrochemical transformation is specifically related to the chemical moieties present in the TCC adhesive, with the pathway of converting TCC‐catechols to quinones appearing most likely for the anodic half‐cell reaction.

To elucidate chemical reactions in adhesive bulk material, FTIR spectroscopy is a commonly used tool, providing insightful analysis. Since an excess of quinones over thiols (1/0.8) was found to boost the bonding strength in the base mixture of IonoBlackTQ, the characteristic quinone vibrational band was present after curing. These unreacted quinones impeded the direct detection of the formation of additional TCC‐quinones during electrochemical debonding. However, the robust applicability of the curing reaction (Figure 3C) allows for the utilization of a substoichiometric amount of quinones, thereby giving the flexibility to fine‐tune the adhesive performance toward specific application requirements. The combination of TQ/ETTMP_700_ in a Q/T ratio of 1/1.1 and the addition of CB and IL (IonoBlackTQ_1.1_) allows for curing that consumes the quinones quantitatively, resulting in the absence of the quinone band in the FTIR‐spectrum measured on the cured adhesive (Figure 4E, black curve). As hypothesized for the electrochemical oxidation of the TCC units, upon voltage application, the formation of an *ortho*‐quinone vibration band at 1664 cm^−1^ was observed, measured at the failed adhesive interface. Interestingly, the characteristic band was pronounced after 5 min already, with the rest of the spectrum remaining constant (Figure 4E). This proves that the TCC oxidation is triggered immediately in the adhesive layer upon voltage application. Subsequently, the band intensity increased only slightly (Figure 4E, full spectra in Figure S25, Supporting Information). The debonding effect observed after 30 min upon applying 9 V proved a significant reduction in the shear strength of this variation of the formulation IonoBlackTQ_1.1_, with a 78% decrease from 7.4 ± 0.4 to 1.6 ± 0.1 MPa (Figure S33, Supporting Information). Due to these parallel observations, this mechanism is also applicable for the best‐performing formulation of IonoBlackTQ. Consistent with the aforementioned decay of the current and the IR‐camera temperature profile, the significant shear strength reduction was evident already after 5 min as well (Figure S37, Supporting Information). This confirms that the majority of quinones are formed early during voltage application. Nevertheless, short voltage application resulted in less clean control of the fracture pattern, indicating overlaying effects on weakened adhesion and cohesion.

These results confirm that electrochemical catechol‐to‐quinone oxidation occurs upon voltage application in the TCC adhesive, enabling debonding. However, two‐electrode CVs (Figure 4D) preclude the separation of electrochemical oxidative and reductive half‐cell reactions. To elucidate which half‐cell reaction occurs most likely to achieve charge neutrality during the debonding oxidation process, three‐electrode CVs on relevant chemical moieties were investigated. Considering that the electrochemical reduction of the imidazolium anion of the IL has been documented before, its role in the present adhesive was questionable.^[^ ^18i^ ^]^ Notably, debonding was found independently in CB or CB+IL filled TQ/ETTMP_700_ adhesives at 9 V, and the two‐electrode CV experiments showed the same features independent of the IL. The CVs revealed that BMIM·NTf_2_ is electrochemically stable in the potential window of the used electrolyte (*N,N*‐dimethylformamide with tetrabutylammonium tetrafluoroborate), while TQ and the thiol ETTMP_700_ can be reduced (Figure S39, Supporting Information). In the two‐electrode CV (Figure 4D), it is expected that initially, the oxidation and reduction with the smallest voltage difference will occur simultaneously at the peak current. Most likely, the oxidation of the TCC catechols was balanced by the reduction of unreacted thiols, newly formed disulfides, or residual TQ. The peak voltage in the two‐electrode CV is larger than the smallest potential differences observed in the three‐electrode CVs, which was linked to polarization effects or an ohmic voltage loss (iR drop) across the adhesive layer. The CVs are recorded on gold, and with the active species free to move in the liquid, while the substrate for the glue is aluminum, and the mobility of the species in the layer is limited. The latter conditions are unfavorable for the electrochemical reaction, thus increasing the overpotential to trigger the faradaic current. Nevertheless, the 9 V required for electrochemical debonding of IonoBlackTQ was comparably small for a structural adhesive.

Although IonoBlackTQ debonded with a clean adhesive fracture pattern, there was no evidence for the TCC catechol oxidation reaction to occur only at one of the substrate‐adhesive interfaces. On the contrary, all results indicate that the oxidation occurs within the first few minutes of voltage application throughout the entire layer, leading to the mixed‐mode fracture pattern as observed in Figure 4B in the absence of IL or after only 5 min voltage application for all formulations. Parallels can be drawn to electrochromic devices, where the species participating in the half‐cell reactions can be mixed in a gel without separating the half‐cells, as it would be required in a battery.^[^ ^30^ ^]^ In this context, it is important to note that the irreversibility of the reaction might be advantageous to prevent the spontaneous re‐reaction at open circuit, re‐establishing the adhesive forces.^[^ ^31^ ^]^ While the TCC catechol oxidation is independent of the presence of IL fillers, such additives impact the debonding process, promoting clean and smooth failure of the adhesive at the cathodic adhesive‐substrate interface, most likely by electromigration. This process is slower than the electrochemical reaction, which is why the clean failure at the cathodic substrate only occurs after 30 min, while the mixed‐failure mode debonding is already possible after 5 min at the same shear strength reduction. Overall, the combination of CB, IL, and TCC adhesive was found to be essential to realize the intended electrochemical debonding mechanism of this structural adhesive through electrochemical oxidation of TCC functionalities, while the analogous epoxy adhesive formulation was not debondable, marking a huge step forward in the field of both mussel‐inspired and electrochemically debondable adhesives by rational design.

**Figure 5:**
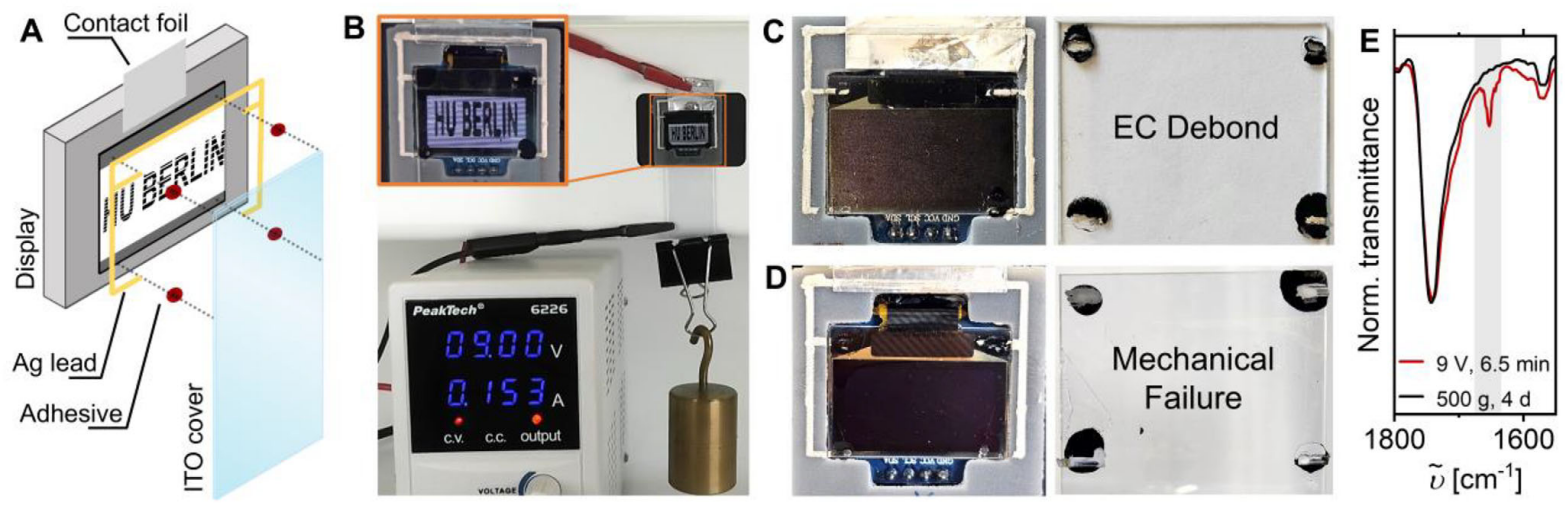
Electrochemical debonding of IonoBlackTQ_1.1_ in a display model system. A) Schematic representation of the mini display model. B) Display–ITO–glass composite system with IonoBlackTQ1.1 under an applied potential of 9 V and a load of 500 g. C) Prototypes (display and cover glass) after debonding by application of 9 V for 6.5 min and D) after mechanical failure following 4 days. E) FTIR spectra of the adhesive with and without electrochemical debonding, normalized to the ester carbonyl band at 1739 cm^−1^.

This adhesive platform has potential for many applications, due to its tunability. With IonoBlackTQ, a structural adhesive of remarkable strength was formulated, which could be suitable for high‐performance applications, e.g. automotive. However, softer formulations like IonoBlackTQ_1.1_ might be applicable in more fragile devices. In order to prove the feasibility of the IonoBlackTQ adhesive family for establishing electrochemical debonding in such microelectronic devices, a concept study was performed by utilizing IonoBlackTQ_1.1_ to reversibly bond a display model cover glass to a 0.96‐inch display (**Figure** **5**A). The replacement of damaged display cover glass is one of the most common repair needs for smartphone devices, the demand of which is constantly increasing due to the trend toward the right to repair and a circular economy.^[^ ^32^ ^]^ To utilize the electrochemical trigger, display contacts were printed in 1 mm line width, using commercially available curable silver ink and a direct‐write ink dispenser to preset four adhesive points positioned in each corner of the display (Figure 5A,B). Indium tin oxide (ITO) cover glass was used, but could potentially be replaced by any conductive glass in a real‐world application scenario.

IonoBlackTQ_1.1_ was manually applied to the corner contacts, and the display's ITO cover glass was positioned using paper backfold clips, resulting in four adhesive points with dot sizes of ≈4 ± 2 mm^2^ (Figure 5B). After curing, the setup yielded a highly robust bond, as confirmed by control samples that withstood a 0.5 kg load for several days (Figure 5D). Upon application of 9 V, the prototype enabled effective and rapid debonding of the glass from the display within 6.5 min under the same load (Figure 5C, Video S1, Supporting Information). A debonding time in the order of five minutes balances efficiency with controlled chemical transformation. Faster release rates might be feasible via higher voltages or loads, but risk side reactions and compromise the substrate material stability in fragile devices. Consistent with the previous shear test results, FTIR spectroscopy of the electrochemically debonded adhesive revealed characteristic quinone vibrations indicative of the formed TCC‐quinone structures, where such features are absent in mechanically detached controls (Figure 5E). The failure analysis revealed that debonding was primarily dominated by the peel off of the adhesive/display interface, as 86% of the effective delamination area consisted of the adhesive/display interface, and only 14% consisted of the ink/display interface. Minor delamination observed in the silver ink could be attributed to suboptimal ink curing (Section S5.10, Supporting Information). The thermal stability of sensitive display components required a reduced curing temperature of the silver ink at 90 °C. Tests on glass/Ag‐ink/IonoBlackTQ_1.1_/Al assemblies, in which the ink was pre‐cured at 120 °C in accordance with the manufacturer's specifications, demonstrated that the silver ink withstands mechanical stress and IonoBlackTQ_1.1_ debonds cleanly, also from the ink regions. Although these findings suggest potential room for optimization, the reduced adhesion of the silver ink on the display was not a limiting factor for electrochemically triggered debonding. For the control samples, bonding failure occurred after 4 days of prolonged mechanical loading with 0.5 kg, with delamination of the silver ink occurring predominantly (Figure 5D). Obviously, mechanical debonding of the IonoBlackTQ adhesive family proceeds at a significantly slower rate than electrochemical debonding, highlighting the potential of tailored adhesive debonding for integration into microelectronic applications.

## 3 Conclusion

A novel bioinspired, solvent‐free two‐component (2K) adhesive system was presented, that combines lignin‐inspired tris‐*ortho*‐quinone (TQ) monomers with star‐polyester trithiol building blocks. In bulk mixtures, the robust thiol‐quinone *Michael*‐type polyaddition proceeds cleanly to form thiol‐catechol connectivities (TCCs), which not only act as network linkers and adhesion promoters but also represent the key functionalities to implement the electrochemical debonding mechanism. The non‐formulated 2K base resin, upon curing, attained a high shear strength of up to 13.8 ± 0.4 MPa on aluminum and demonstrated the potential for controlled debonding under voltage application. By incorporating small amounts of conductive additives such as carbon black (CB) and an ionic liquid (BMIM·NTf_2_), the IonoBlackTQ formulation was obtained. This could be cured to a structural adhesive that reaches strengths of up to 19.6 ± 0.7 MPa and enabled clean electrochemical debonding, which significantly reduces the adhesive strength after voltage application by 72–86%. The application of 9 V for 30 min not only significantly reduces the adhesive strength but also results in a purely adhesive fracture pattern of the fully formulated adhesive, allowing clean and efficient removal of the adhesive from the cathodic substrate. Interestingly, evidence from CV and IR analysis indicates that the Faradaic electrochemical transformation is largely completed within ≈5 min at 9 V. A transfer of the conductive formulation additives of IonoBlackTQ system to a commodity 2K epoxy adhesive revealed that the electrochemical debonding capability originates from the TCC‐based adhesive, while the additives only facilitate the fracture pattern and support the conductivity. The oxidation of the TCC‐functionalities occurs in the entire bulk of the adhesive, not only changing properties at the adhesive interface. Ultimately, the debonding of a tightly bonded cover model glass from a 1‐inch display on electrochemical command has been demonstrated by triggering the debonding of the IonoBlackTQ adhesive family with 9 V. The detachment of the cover glass occurred rapidly within 6.5 min under a 0.5 kg load, making the variations of the structural adhesive IonoBlackTQ a promising solution for advanced repair and recycling strategies with potential in different fields of application.

Supporting Information is available from the Wiley Online Library or from the author. Additional references were used in Supporting Information.^[^ ^33^, ^34^, ^35^, ^36^, ^37^, ^38^, ^39^, ^40^, ^41^ ^]^

## Conflict of Interest

The authors declare no conflict of interest.

## Supporting information



Supporting Information

Supplemental Video 1

## Data Availability

The data that support the findings of this study are available from the corresponding author upon reasonable request.
