# Supplementary material for: Unlocking the Essence of Lignin: High‐Performance Adhesives That Bond via Thiol‐Catechol Connectivities and Debond on Electrochemical Command
Source: Adv Mater. 2025 Aug 15;37(43):e10463. doi: 10.1002/adma.202510463 (PMC12574627; doi:10.1002/adma.202510463)
Supplement: Supplementary file 1 — Supporting Information [file ADMA-37-e10463-s001.pdf]

ADVANCED MATERIALS

Supporting Information

for *Adv. Mater.*, DOI 10.1002/adma.202510463

Unlocking the Essence of Lignin: High-Performance Adhesives That Bond via Thiol-Catechol Connectivities and Debond on Electrochemical Command

Keven Walter, Dominik P. Hoch, Leon Hertweck, Kannan Balasubramanian, Jonas Geisler, Mathias Röllig, Tilmann J. Neubert and Hans G. Börner**

Supporting Information

Unlocking the Essence of Lignin: High-Performance Adhesives that Bond via Thiol-Catechol Connectivities and Debond on Electrochemical Command

Keven Walter, Dominik P. Hoch, Leon Hertweck, Kannan Balasubramanian, Jonas Geisler, Mathias Röllig, Tilmann J. Neubert and Hans G. Börner**

K. Walter, D. Hoch, L. Hertweck, Prof. Dr. K. Balasubramanian, Dr. Jonas Geisler, Dr. T. J. Neubert, Prof. H. G. Börner; Humboldt-Universität zu Berlin, Institute of Chemistry, Unter den Linden 6, 10117 Berlin, Germany

Dr. T. J. Neubert; Friedrich-Schiller-Universität Jena, Institute for Technical and Environmental Chemistry, Philosophenweg 7a, 07743 Jena, Germany

L. Hertweck, Prof. Dr. K. Balasubramanian; Humboldt-Universität zu Berlin, School of Analytical Sciences Adlershof, Unter den Linden 6, 10117 Berlin, Germany

M. Röllig; Bundesanstalt für Materialforschung und –prüfung (BAM), 8.3 Thermografische Verfahren, Richard-Willstätter-Straße 11, 12489 Berlin

Table of contents

S1 Chemicals, Solvents and Material ... 3
S2 Instrumentation ... 5
S3 Synthesis and Analysis ... 7
S3.1 Synthesis of *mini*-lignin ... 7
S3.2 Demethylation of *mini*-lignin ... 8
S3.4 Synthesis of TQ ... 9
S3.4.1 Fully oxidized TQ ... 9
S3.4.2 Scalable TQ Synthesis ... 11
S3.5 Synthesis of *mini*-PEG thiol ... 13
S3.5.1 Synthesis of *para*-toluenesulfonyl triethylene glycol monomethyl ether ... 13
S3.5.2 Synthesis of *mini*-PEG thiol ... 14
S4 Experimental procedures and results ... 15
S4.1 IBX-mediated oxidative *O*-demethylation of *mini*-lignin ... 15
S4.2 Oxidation of demethylated *mini*-lignin ... 17
S4.3 Thermal stability testing of TQ ... 19
S4.4 Determining the reactivity in solution ... 20
S4.5 Hydrolysis of 2K System ... 22
S4.6 Preparation lap shear testing with debonding ... 23
S4.6.1 Lap shear testing ... 23
S4.6.2 Debonding procedure ... 23
S4.6.3 Solvent robustness testing procedure ... 23
S4.7 Statistical Analysis ... 24
S5 Experimental Data ... 24
S5.1 Characterization of the thiols ... 24
S5.2 Temperature-dependent oscillatory tests *via* rheology ... 24
S5.3 Isothermal experiments rheology ... 26
S5.4 FTIR analysis ... 26
S5.4.1 Transition from THPE to TQ ... 26
S5.4.2 Curing behavior of TQ with *mini*-PEG-SH with different curing conditions ... 27
S5.4.3 TQ + trithiols (Q/T, 1/1) ... 28
S5.4.4 TQ + $ETTMP_{700}$ (Q/T, 1/0.8) ... 29
S5.4.5 Analysis after Debonding experiment ... 30
S5.5 Thermogravimetric analysis (TGA) ... 31
S5.6 Differential scanning calorimetry (DSC) ... 32
S5.6.1 DSC analysis of non-cured adhesive mixtures ... 32
S5.6.2 DSC analysis of cured adhesive mixtures ... 32
S5.7 Lap Shear Testing ... 34
S5.7.1 Temperature-dependent curing (60 °C/90 °C) ... 34
S5.7.2 TQ/$ETTMP_{700}$ (1/0.8) + CB + IL (incl. debonding) ... 35
S5.7.3 TQ/$ETTMP_{700}$ (1/1.1) + CB + IL (incl. debonding) ... 35
S5.7.4 Fracture pattern analysis ... 36
S5.7.5 Substrate compatibility of IonoBlackTQ ... 36
S5.7.6 Solvent resistance testing ... 37
S5.8 Thermographic analysis ... 37
S5.9 Cyclic voltammetry (CV) ... 39
S5.10 Mini display model study ... 41
S5.10.1 Preliminary experiments ... 41
S5.10.2 Building the mini display model ... 42
S5.11 Investigation of fracture pattern surface morphology ... 42
S6 References ... 43

S1 Chemicals, Solvents and Material

Chemicals

Boron tribromide (>99%, Sigma Aldrich, Darmstadt, Germany), 1-butyl-3-methylimidazolium bis(trifluoromethylsulfonyl)imide (BMIM·NTf_2, 99%, abcr GmbH, Karlsruhe, Germany), carbon black (Denka Chemicals Holdings Asia Pacific Pte Ltd), ethanethiol (>98%, Tokyo Chemical Industry Co., LTD., Tokyo, Japan), (diacetoxyiodo)benzene (PIDA, 98%, abcr GmbH, Karlsruhe, Germany), ethoxylated trimethylolpropane tri (3-mercapto-propionate) (THIOCURE®333, $ETTMP_{1300}$ or THIOCURE®332, $ETTMP_{700}$, Bruno Bock Chemische Fabrik GmbH & Co. KG, Marschacht, Germany), formic acid (FA, 98+%, Thermo Fisher Scientific, Darmstadt, Germany), guaiacol (98%, Tokyo Chemical Industry Co., LTD., Tokyo, Japan), hydrochloric acid (37%, Grüssing GmbH, Filsum, Germany), 2-iodobenzoic acid (98%, Tokyo Chemical Industry Co., LTD., Tokyo, Japan), OXONE® (Thermo Fisher Scientific, Kandel, Germany), magnesium sulfate hydrate (99%, Sigma Aldrich, Darmstadt, Germany), *para*-toluene sulfonic acid monohydrate (Sigma Aldrich, Darmstadt, Germany), *para*-toluene sulfonic acid monohydrate (>98%, Sigma Aldrich, Darmstadt, Germany), sodium bicarbonate (99%, Carl Roth GmbH + Co. KG, Karlsruhe, Germany), Sodium hydrosulfide (>70%, Carl Roth GmbH + Co. KG, Karlsruhe, Germany), sodium hydrosulfite (85%, abcr GmbH, Karlsruhe, Germany) sodium periodate (99.8+%, Thermo Fisher Scientific, Kandel, Germany and 99.8+%, Sigma-Aldrich, Darmstadt, Germany), triethylamine (99.5%, Sigma Aldrich, Darmstadt, Germany), triethylene glycol monomethyl ether (>98%, Tokyo Chemical Industry Co., LTD., Tokyo, Japan), 1,1,1-tris(4-hydroxyphenyl)ethane (THPE, 99%, abcr GmbH, Karlsruhe, Germany), 1,1,1-tris(hydroxymethyl)ethane tris(3-mercaptopropionate) (TTMP, >95%, Sigma Aldrich, Darmstadt, Germany), vanillin (>98%, Tokyo Chemical Industry Co., LTD., Tokyo, Japan), zinc chloride (>98%, Alfa Aesar, Haverhill, Massachusetts, USA).

Iodoxybenzoic acid (IBX) was synthesized in accordance to Frigerio *et al.*[1] as previously described.[2] CAUTION! IBX is explosive under heating >200 °C or impact![3]

Solvents

Dimethylformamide (DMF, peptide grade, VWR chemicals (Dresden, Germany), dry dichloromethane (DCM, 99.9%, ExtraDry, Thermo Fisher Scientific, Darmstadt, Germany), N-Methyl-2-pyrrolidone (NMP, peptide-grade, IRIS Biotech GmbH Marktredwitz, Germany), tetrahydrofurane (THF, HPLC-grade, VWR International GmbH, Rosny-sous-Bois, France)

and acetonitrile (LC-MS grade, VWR International GmbH, Rosny-sous-Bois, France) were used as received.

Acetone, dichloromethane, chloroform, ethyl acetate, isopropanol and methanol (technical grade, Stockmeier Chemie GmbH, Bielefeld, Germany) were distilled prior to use.

Ultrapure water was produced using a SG LaboStar® TM 1-UV system from SG water (Hamburg, Germany). An Evoqua Water Technologies Polisher HP2 module was used as the ion exchanger. The electrical conductivity of the ultrapure water was 0.055 $\mu S \cdot cm^{-1}$.

For NMR spectroscopy, DMSO-d_6 (D >99.8%), and $CDCl_3$ (D >99.8%) from Deutero GmbH (Kastellaun, Germany) were used.

Materials

Aluminum, steel and glass substrates (20 mm x 80 mm x 2 mm) for shear testing have been purchased from Rocholl GmbH (Eschelbronn, Germany).

Indium tin oxide glasses (ITO) were purchased from Ossila (Leiden, the Netherlands).

As 2K epoxy adhesive UHU Plus Schnellfest (UHU GmbH & Co.KG, Bühl, Germany) have been used.

For the thermographic experiments, a paint (HEWIP-LT-MQIR-BK-11) from Lab IR Paints (Plzeň, Czech Republic) was used to ensure high and homogeneous emissivity.

0.96-inch OLED displays (SSD1306 Display I2C 128 x 64) from AZ Delivery (Deggendorf, Germany) was used for the prototypes.

The conductive ink used was a silver ink (Conductor 3) from Voltera (Waterloo, Canada).

As conductive adhesive tape an aluminum-based tape (TRU COMPONENTS AL-F2.5R1000 ESD-Klebeband) form Conrad Electronic SE (Hirschau, Germany) was utilized.

S2 Instrumentation

For shear strength testing an extensometer Texture Analyzer Ta.XT.plus100C (Stable Micro Systems, Godalming, United Kingdom) with a 100 kg force cell or a 34TM-30 universal testing machine (Instron GmbH, Darmstadt, Germany) and a 25000 N load cell has been utilized.

Fourier transform infrared spectroscopy (FTIR) measurements were carried out on a Bruker Vertex 70v FT-IR spectrometer (Bruker Optik GmbH, Ettlingen, Germany). Blank measurements were conducted before and after each sample.

Gel permeation chromatography (GPC) measurements were carried out on an Eco-SEC-System with UV and RI-detection (HLC-8320 GPC) from Tosoh (Griesheim, Germany). As solvent tetrahydrofuran (THF, HiPerSolv CHROMANORM® for HPLC) from VWR® Chemicals (Dresden, Germany) was used and SDV columns (1000 Å 5 µm, 100000 Å 5 µm and 1000000 Å 5 µm) from PSS (Mainz, Germany) were applied. The molar mass and dispersity values were calculated against polystyrene standards (Agilent Technologies: M_p = 580, 4730, 12980, 19920, 110 000 g/mol; MACHEREY-NAGEL GmbH & Co. KG: 1060, 2950, 9200, 30.3 k, 66 k, 220 k, 514 k, 1950 k, 3040 k g·mol^{-1}) and polyethylene glycol standards (PSS Polymer Standard Service GmbH; M_p = 194, 430, 1030, 2130, 3450, 6530, 11.4 k, 25.3 k, 44 k g·mol^{-1}).

For bladeless shear mixing a SpeedMixer DAC 150 SP (Hauschild, Hamm, Germany) has been utilized. All mixtures were homogenized with progressively increasing mixing rate. The following program has been utilized: 1500 rpm (20 s), 2000 rpm (20 s), 2500 rpm (20 s), 3000 rpm (40 s) and 3500 rpm (60 s).

Nuclear magnetic resonance spectroscopy (NMR) measurements were performed on a Bruker Avance II 300 MHz, Avance 400 MHz or Avance III 500 MHz spectrometer (Bruker BioSpin GmbH, Rheinstetten, Germany) in the given deuterated solvent.

Electrochemical debonding between the substrates was performed using a PeakTech® P 6226 switching mode power supply (0 - 30 V / 0 - 10 A, PeakTech Prüf- und Messtechnik GmbH, Ahrensburg, Germany) at the specified voltages or currents.

Two-electrode cyclic voltammetry measurements in solid state were performed with a BP-300 (Biologic, Seyssinet-Pariset, France).

Cyclic voltammetry measurements were performed in solution using an EmStat4S (PalmSens BV, Houten, the Netherlands). A silver wire was used as the quasi-reference electrode, a gold disk electrode as the working electrode, and a platinum wire as the counter electrode.

Differential scanning calorimetry (DSC) analysis was performed on a DSC 3 (Mettler-Toledo GmbH, Gießen, Germany) connected to a cooler TC100 (Peter Huber Kältemaschinenbau SE, Offenburg, Germany). All measurements were performed under an argon atmosphere.

Rheology measurements were carried out on an MCR 302e (Anton Paar GmbH, Graz, Austria) rheometer with a plate/plate (PP) measuring geometries with disposable-plate measuring system and gap width of 0.5 mm.

Printing for the mini display model was performed using a V-One Printer (Voltera, Waterloo, Kanada). Thermal gravimetric analysis (TGA) measurements were carried out on a Thermogravimetric Analyzer Pyris 1 (Perkin Elmer, Waltham, USA). Approximatively 10 mg of each sample were weighed in a ceramic pan and heated from 30 °C up to 800 °C at a heating rate of 20 °C/min, under argon atmosphere with a flow rate of 20 mL/min.

Ultra-high-performance liquid chromatography with electrospray ionization (ESI) mass spectrometry (UHPLC) was carried out on an ACUIDITY-UPLC® H-Class CM Core System of Waters GmbH (Eschborn, Germany). Detection was done utilizing an ACUIDITYUPLC® photo diode array (PDA)-detector (wavelength range 190-500 nm) and an ACUIDITY-UPLC® QDa mass detector with ESI. For analysis Waters software EmpowerTM3 was used. Separation was conducted with ACUIDITY-UPLC® BEH C18 VanGuardTM precolumn (110 Å, 1.7 µm, 5 × 21 mm ID) and an ACUIDITY-UPLC® BEH C18-column (110 Å, 1.7 µm, 5×21 mm ID) from Waters. As mobile phase, mixtures of solvent A (Milli-Q water with 0.1% FA, v/v) and solvent B (acetonitrile with 0.1% FA, v/v) were used with 0.5 mL·min^{-1} flow rates.

UV-visible spectroscopy (UV/vis)_measurements were performed on a Spectrometer UV-2501PC from Shimadzu Deutschland GmbH, Duisburg, Germany. The recorded spectra ranged from 200 to 800 nm.

Scanning electron microscopy (SEM) images were measured on a *JEOL JCM-6000* in high vacuum with 15 kV accelerating voltage.

S3 Synthesis and Analysis

S3.1 Synthesis of *mini*-lignin

Vanillin (10.0 g, 65.7 mmol, 1 eq.) was dissolved in guaiacol (36.8 mL, 328.6 mmol, 5 eq.) before zinc chloride (0.9 g, 6.6 mmol, 0.1 eq.) and *para*-toluene sulfonic acid (1.1 g, 6.6 g, 0.1 eq.) were added. The mixture was stirred at 50 °C for 5 d before it was precipitated into chloroform. The solids were collected *via* filtration and washed with an aqueous methanol solution (5x 100 mL, 10%). The obtained product was dried under reduced pressure at 110 °C, yielding a red powder (9.1 g, 23.8 mmol, 40%).

^{1}H NMR (DMSO-d$_6$, 500 MHz) δ [ppm] = 8.80 (s, 3H), 6.81 – 6.62 (m, 6H), 6.48 (dd, *J* = 8.2, 1.8, 3H), 5.27 (s, 1H), 3.66 (s, 9H).
^{13}C NMR (DMSO-d$_6$, 126 MHz): δ [ppm] = 147.2, 144.7, 135.7, 121.2, 115.1, 113.3, 79.2, 55.6, 54.7.
UHPLC-ESI-MS t_R = 2.31 min (λ = 280 nm, gradient 10-90% B with A: H_2O + 0.1% FA and B: ACN + 0.1% FA); found (m/z): 259.36 $[M+H\text{-guaiacol}]^+$ 382.4 $[M+H]^+$, calculated (m/z): 259.10 $[M+H\text{-guaiacol}]^+$, 382.1 $[M+H]^+$.
FTIR (ATR): $\tilde{\nu}$ [cm^{-1}] = 3387 (s), 2977 (s), 2859 (m), 1614 (w), 1503 (s), 1452 (m), 1427 (m), 1370 (w) 1264 (m), 1240 (m), 1200 (s), 1148 (m), 1120 (m), 1034 (m), 749 (m), 639 (w), 551 (w).

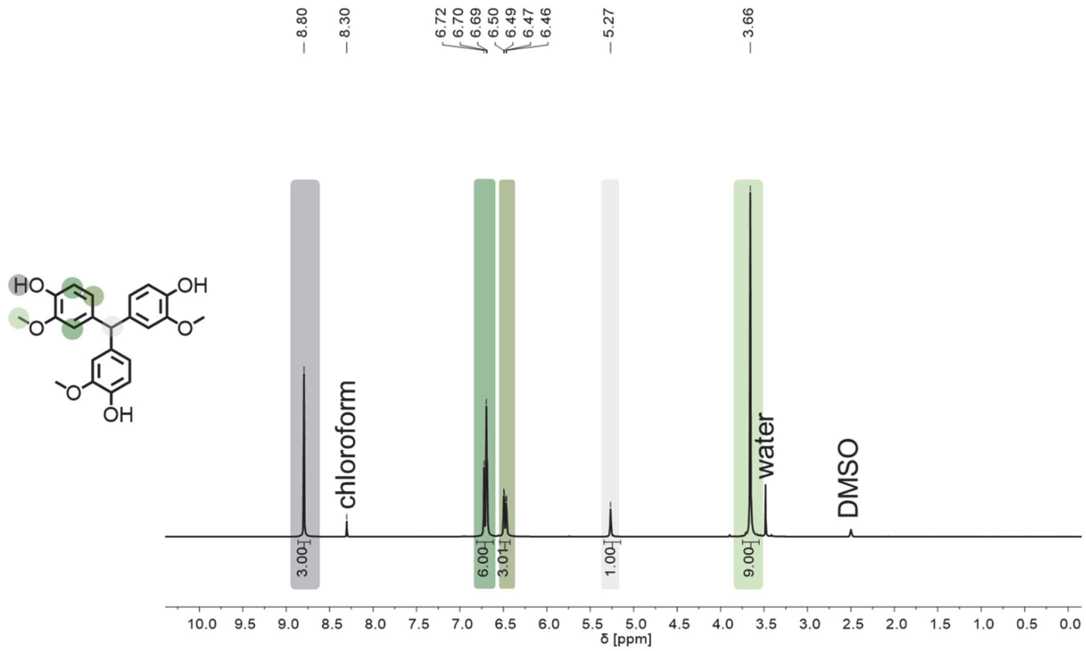

Figure S1. ^{1}H NMR of *mini*-lignin in DMSO-d_6.

S3.2 Demethylation of *mini*-lignin

The demethylation was performed following the protocol from Vickery *et. al.*.[4] The reaction was conducted under an inert atmosphere using dry glassware.

A solution of *mini*-lignin (250 mg, 0.65 mmol, 1 eq.) in dry dichloromethane (20 mL) was cooled to -84 °C before adding a solution of boron tribromide (2.5 mL, 2.5 mmol, 1 M) in dichloromethane. The reaction mixture was stirred for 2 h at -84 °C before it was allowed to heat up to room temperature, where it was stirred for an additional 3 h. Then methanol was slowly added before the mixture was dried under reduced pressure. The raw product was purified *via* reverse phase chromatography (water/acetonitrile, 80/20, v/v). The obtained product fractions were freeze dried, yielding demethylated *mini*-lignin as red powder (172 mg, 0.51 mmol, 78%).

^{1}**H NMR** (DMSO-d$_6$, 500 MHz) δ [ppm] = 8.67 (s, 5H), 6.61 (d, *J* = 8.1 Hz, 3H), 6.50 – 6.41 (m, 3H), 6.31 (dd, *J* = 8.2, 2.0 Hz, 3H), 5.00 (s, 1H).
^{13}**C NMR** (DMSO-d$_6$, 126 MHz): δ [ppm] = 145.1, 143.7, 136.5, 120.2, 116.9, 115.5, 54.6.
UHPLC-ESI-MS t_R = 1.60 min (λ = 280 nm, gradient 10-90% B with A: H_2O + 0.1% FA and B: ACN + 0.1% FA); found (m/z): 341.38 $[M+H]^+$, calculated (m/z): 341.10 $[M+H]^+$.
FTIR (ATR): $\tilde{\nu}$ [cm^{-1}] = 3377 (s), 2702 (w), 1608 (m), 1510 (s), 1440 (m), 1357 (m), 1271 (s), 1243 (s), 1188 (s), 1107 (m), 975 (m), 933(w), 875 (m), 823 (w), 754 (m), 644 (w).

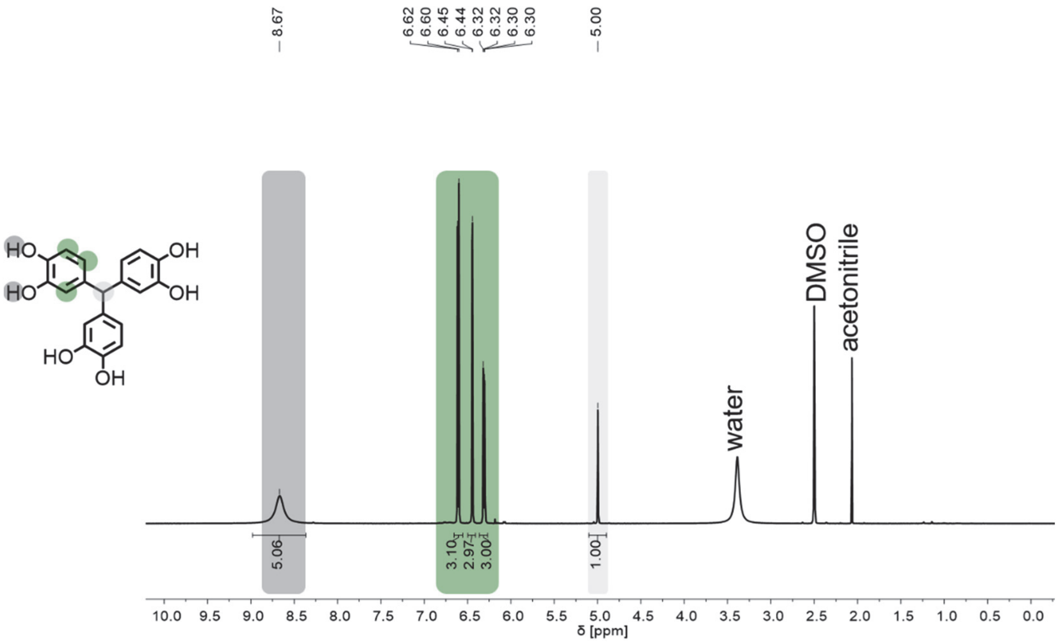

Figure S2. ^{1}H NMR of demethylated *mini*-lignin in DMSO-*d*$_6$.

S3.4 Synthesis of TQ

The synthesis of trisquinone (TQ) is divided into two parts as described in the main manuscript. First, the synthesis was carried out according to the protocol known from the literature,[2, 5] which led to a fully oxidized TQ (Section 3.4.1). However, it should be noted that the production is very laborious and therefore scalable only to a limited extent.
In an optimized production protocol, upscaling was achieved by accepting a triphenol that was not fully chemically converted (Section 3.4.2). This resulted in a conversion of 94% of all phenols leading to the formation of a bisquinone with 17% as a by-product.

S3.4.1 Fully oxidized TQ

The synthesis of fully oxidized TQ was divided into two steps. First, 1,1,1-tris(4-hydroxyphenyl) ethane (THPE) was oxidized with an excess of 2-iodoxybenzoeic acid (IBX) and then reduced. The resulting triscatechol was isolated by column chromatography and then oxidized back to triscatechol. Resulting in a total yield of 31%.

A) Synthesis of 4,4',4''-(ethane-1,1,1-triyl)tris(catechol)

THPE (5.0 g, 17.1 mmol, 1 eq.) was dissolved in methanol (200 mL) before IBX (27.4 g, 97.9 mmol, 2.94 eq.) was added. The reaction mixture was stirred for 15 min at room temperature and then sodium hydrosulfite (85%, 21.2 g) and ultrapure water (200 mL) were added. Again, the reaction mixture was stirred for 15 min at room temperature, before it was extracted three times with ethyl acetate. After drying the combined organic phases over magnesium sulfate and removing the solvent under reduced pressure the raw product was purified *via* reverse phase chromatography (water/acetonitrile, 75/25, v/v). The obtained product fractions were freeze dried yielding 4,4',4''-(ethane-1,1,1-triyl)tris(catechol) as beige foam (4.1g, 11.5 mmol, 67%).

^{1}H NMR (500 MHz, DMSO-d_6) δ [ppm] = 8.67 (s, 1H), 8.63 (s, 1H), 6.60 (d, J = 8.3 Hz, 1H), 6.42 (d, J = 2.3 Hz, 1H), 6.28 (dd, J = 8.2, 2.3 Hz, 1H), 1.88 (s, 1H).
^{13}C NMR (126 MHz, DMSO-d_6) δ [ppm] = 144.5, 143.4, 141.4, 119.5, 116.8, 115.0, 50.4, 31.0.
UHPLC-ESI-MS t_R = 1.76 min (λ = 280 nm, gradient 10-90% B with A: H_2O + 0.1% FA and B: ACN + 0.1% FA); found (m/z): 353.21 $[M-H]^-$, calculated (m/z): 353.10 $[M-H]^-$.
FTIR (ATR): $\tilde{\nu}$ [cm^{-1}] = 3324 (s), 1601 (m), 1515 (s), 1424 (m), 1329 (w), 1279 (s), 1249 (s), 1105 (m), 936 (m), 918 (w), 871 (m), 816 (w), 765 (m), 640 (w), 592 (w).

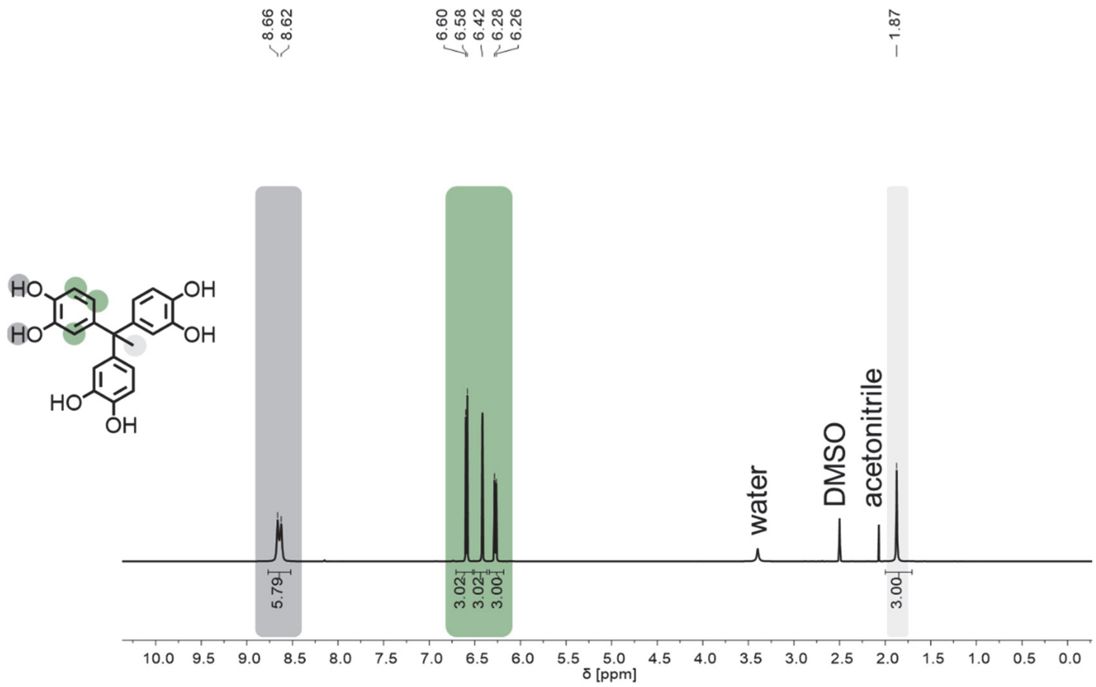

Figure S3. ^{1}H NMR of triscatechol in DMSO-d_6.

B) Oxidation of 4,4',4''-(ethane-1,1,1-triyl)tris(catechol)

4,4',4''-(Ethane-1,1,1-triyl)tris(catechol) (500 mg, 1.41 mmol, 1 eq.) was dissolved in NMP (5 mL) before (diacetoxyiodo)benzene (1363 mg, 4.23 mmol, 3.3 eq.) was added. The mixture was stirred at room temperature for 30 min, before it was precipitated into cold methanol. The mixture was then filtered, and the precipitate was washed with cold methanol (3x 10 mL). The obtained product was dried under reduced pressure yielding a red powder (232 mg, 0.67 mmol, 47%).

^{1}H NMR (500 MHz, DMSO-d_6) δ [ppm] = 7.16 (dd, J = 10.4, 2.5 Hz, 3H), 6.48 – 6.41 (m, 6H), 1.80 (s, 3H).
^{13}C NMR (126 MHz, DMSO-d_6) δ [ppm] = 180.0, 179.8, 151.8, 139.6, 130.8, 130.2, 52.6.
UHPLC-ESI-MS t_R = 2.12 min (λ = 280 nm, gradient 10-90% B with A: H_2O + 0.1% FA and B: ACN + 0.1% FA); found (m/z): 349.2 $[M-H]^-$, 699.2 $[2M-H]^-$, calculated (m/z): 347.1 $[M-H]^-$, 696.2 $[2M-H]^-$.
The difference between the calculated and the detected mass is probably due to redox reactions in the ESI source,[6] where *ortho*-quinones are partially reduced to catechols during ionization. In addition, physical dimers can be formed in the ionization process, which cannot be detected by NMR.[7]

FTIR (ATR): $\tilde{\nu}$ [cm^{-1}] = 3068 (w), 1695 (m), 1661 (s), 1618 (m), 1560 (m), 1510 (w), 1458 (w), 1402 (m), 1273 (m), 1175 (w), 1126 (m), 1028 (w), 920 (w), 842 (w), 816 (m), 696 (w), 640 (w).

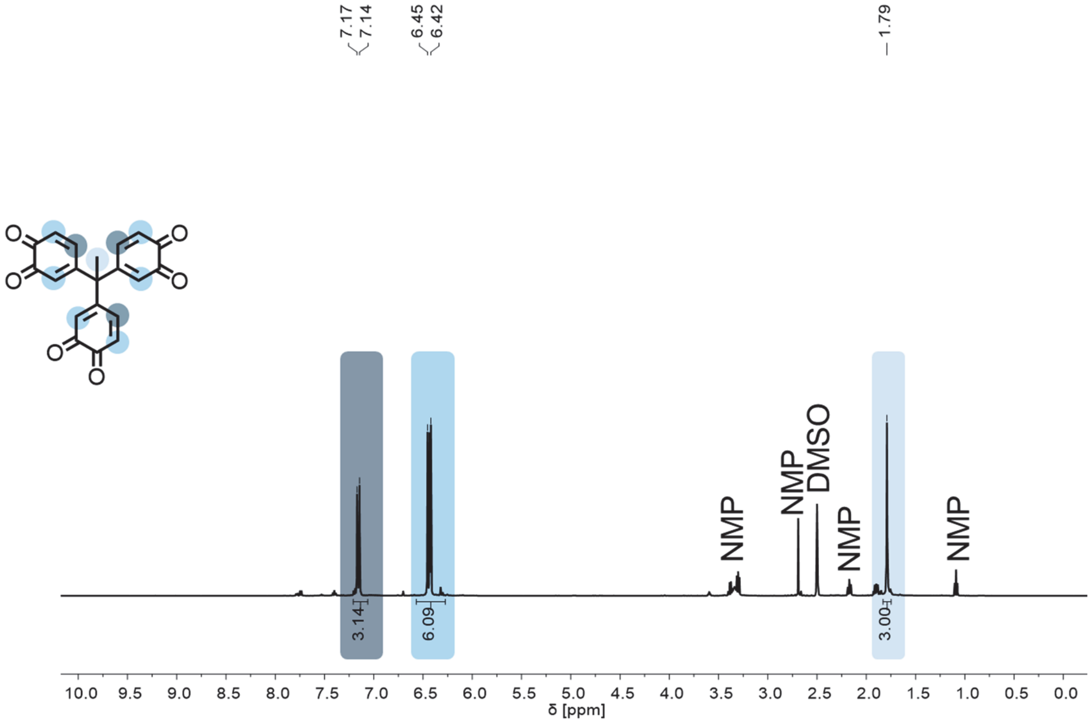

Figure S4. ^{1}H NMR of oxidized triscatechol in DMSO-d_6.

S3.4.2 Scalable TQ Synthesis

THPE (10.0 g, 34.2 mmol, 1 eq.) was dissolved in methanol (200 mL) before IBX (27.4 g, 97.9 mmol, 2.94 eq.) was added. The mixture was stirred at room temperature for 20 min, before it was cooled in an ice bath for another 10 min. The mixture was then filtered, and the precipitate was washed with cold methanol (3x 60 mL). The obtained product was dried under reduced pressure yielding a red powder (8.9 g, 25.6 mmol, 75%).

^{1}H NMR (500 MHz, DMSO-d_6) δ [ppm] = 9.64 (s, 1H), 7.26–7.10 (m, 7H), 7.02 (dd, J = 10.4, 2.4, 1H), 6.79 (d, J = 8.7, 1H), 6.49–6.31 (m, 14H), 6.09 (d, J = 2.2, 1H), 1.82 (d, J = 17.3, 6H).
^{13}C NMR (126 MHz, DMSO-d_6) δ [ppm] = 180.3, 180.1, 180.0, 179.8, 157.5, 155.9, 151.8, 140.7, 139.6, 130.8, 130.3, 130.2, 130.0, 128.2, 116.2, 116.1, 52.6, 52.2, 49.1, 40.5, 40.5, 40.4, 40.3, 40.2, 40.1, 40.0, 39.8, 39.6, 39.5, 23.1, 20.8.
UHPLC-ESI-MS (λ = 280 nm, gradient 10-90% B with A: H_2O + 0.1% FA and B: ACN + 0.1% FA); t_R = 1.37 min found (m/z): 347.17 [M-H]$^-$, calculated (m/z): 347.06 [M-H]$^-$; t_R = 1.74 min found (m/z): 349.15 [M-H]$^-$, 699.17 [2M-H]$^-$ calculated (m/z): 349.07 [M-H]$^-$, 699.15 [2M-H]$^-$; t_R = 1.99 min found (m/z): 333.20 [M-H]$^-$, calculated (m/z): 333.08 [M-H]$^-$.

The difference between the calculated and the detected mass is probably due to redox reactions in the ESI source,[6] where *ortho*-quinones are partially reduced to catechols during ionization. In addition, physical dimers can be formed in the ionization process, which cannot be detected by NMR.[7]

FTIR (ATR): $\tilde{\nu}$ [cm^{-1}] = 3070 (m), 1695 (m), 1662 (s), 1622 (m), 1564 (m), 1512 (w), 1461 (w), 1402 (m), 1382 (m), 1315 (w), 1272 (m), 1178 (w), 1126 (m), 1026 (m), 923 (w), 815 (m), 700 (w), 638 (m), 611 (m).

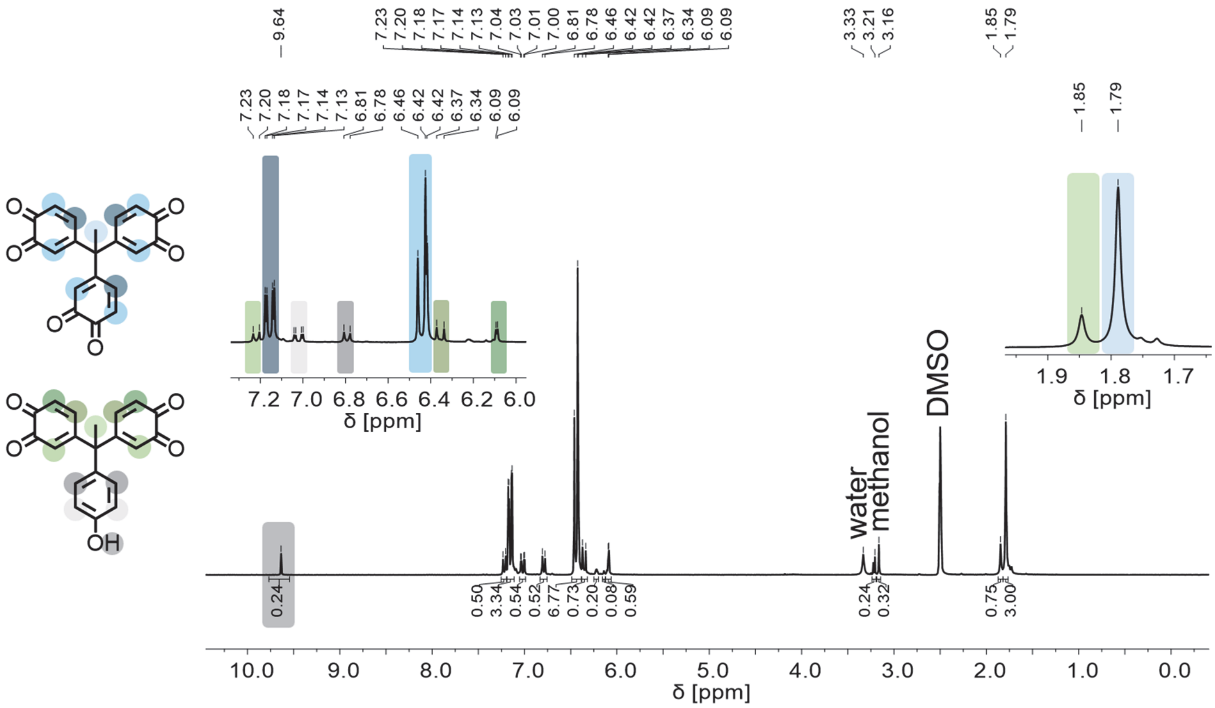

Figure S5. ^{1}H NMR of TQ in DMSO-d_6.

Due to the fact that a sub-stoichiometric amount of IBX was used during the TQ synthesis a full oxidation to a trimeric *ortho*-quinone is not forced. It has been observed *via* ^{1}H NMR that a minor part of the product is only twice oxidized THPE. This observation has been confirmed *via* UHPLC measurements. Both measurements enable a quantification of this byproduct. For quantification *via* ^{1}H NMR the ratio between the benzylic methyl group around δ = 1.83 ppm has been calculated, resulting in 83% trisquinone and 17% bisquinone.

S3.5 Synthesis of *mini*-PEG thiol

The synthesis of the *mini*-PEG thiol was performed in two steps.

S3.5.1 Synthesis of *para*-toluenesulfonyl triethylene glycol monomethyl ether

The tosylation of *mini*-PEG-OH was performed following the protocol from Zhang *et. al.*.[8] Triethylene glycol monomethyl ether (55.0 g, 335 mmol, 1 eq.) and *para*-toluenesulfonyl chloride (70.3 g, 369 mmol, 1.1 eq.) were dissolved in dichloromethane (400 mL). Triethylamine (102 mL, 737 mmol, 2.2 eq.) was added and the solution was stirred at ambient temperature for 3 h, before the resulting suspension was neutralized with concentrated hydrochloric acid (12 M, 61.4 mL). The organic phase was washed with a saturated $NaHCO_3$ solution three times, dried over $MgSO_4$ and the solvent was removed under reduced pressure to obtain *para*-toluenesulfonyl triethylene glycol monomethyl ether (111 g, quantitative) as a colorless oil, which was used without further purification in the next reaction.

^{1}H NMR (500 MHz, $CDCl_3$) δ [ppm] = 7.79 (d, J = 8.3, 2H), 7.36 – 7.31 (m, 2H), 4.18 – 4.13 (m, 2H), 3.70 – 3.66 (m, 2H), 3.62 – 3.57 (m, 6H), 3.55 – 3.50 (m, 2H), 3.37 (s, 3H), 2.44 (s, 3H).

^{13}C NMR (126 MHz, $CDCl_3$) δ [ppm] = 144.9, 133.1, 130.4, 129.9, 128.1, 127.2, 77.4, 77.2, 76.9, 72.0, 70.9, 70.7, 70.7, 69.4, 68.8, 59.2, 21.8.

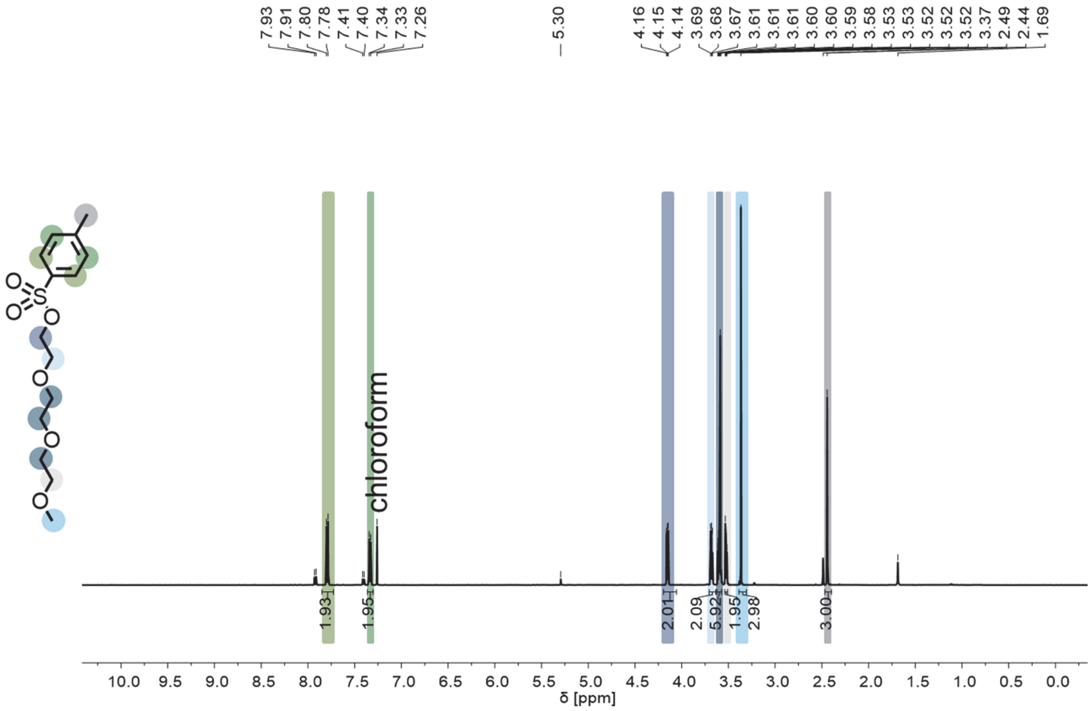

Figure S6. ^{1}H NMR of *para*-toluenesulfonyl triethylene glycol monomethyl ether in $CDCl_3$.

S3.5.2 Synthesis of *mini*-PEG thiol

The synthesis was performed following the protocol from Mahou *et. al.*.[9]

para-Toluenesulfonyl triethylene glycol monomethyl ether (111 g, 335 mmol, 1 eq.) was dissolved in methanol (300 mL) and degassed with argon. Sodium hydrosulfide (93.9 g, 1.68 mol, 5 eq.) was dissolved in water (300 mL) and degassed with argon. The methanolic solution was added to this aqueous solution over the course of 30 min under an argon atmosphere and the resulting mixture was stirred at ambient temperature overnight. The solution was acidified using concentrated hydrochloric acid (12 M, 140 mL) and extracted with dichloromethane three times. The combined organic phase was dried over magnesium sulfate and the solvent removed under reduced pressure. The crude product was further purified by vacuum distillation to obtain *mini*-PEG thiol (29.8 g, 165 mmol, 49%) as a colorless oil.

^{1}H NMR (300 MHz, $CDCl_3$) δ [ppm] = 3.70 – 3.50 (m, 10H), 3.36 (s, 3H), 2.68 (dt, *J* = 8.2, 6.5, 2H), 1.57 (t, *J* = 8.2, 1H).

^{13}C NMR (75 MHz, $CDCl_3$) δ [ppm] = 77.6, 77.2, 76.7, 73.0, 72.0, 70.7, 70.3, 59.2, 24.4.

FTIR (ATR): $\tilde{\nu}$ [cm^{-1}] = 3474 (w), 3460 (w), 3445 (w), 3427 (w), 3404 (w), 3396 (w), 3373 (w), 3356 (w), 3342 (w), 3319 (w), 3310 (w), 3300 (w), 3275 (w), 3263 (w), 3254 (w), 3242 (w), 3227 (w), 3219 (w), 3205 (w), 3178 (w), 2920 (m), 2874 (m), 1695 (w), 1663 (w), 1622 (w), 1566 (w), 1472 (w), 1458 (w), 1404 (w), 1352 (w), 1283 (w), 1101 (s), 1026 (w), 845 (w), 816 (w).

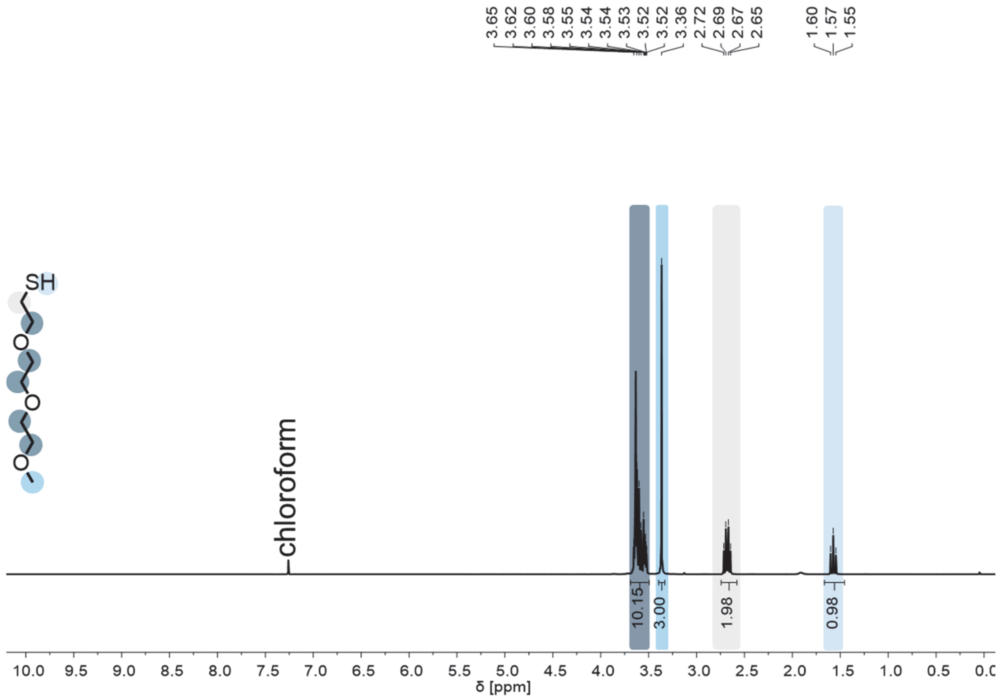

Figure S7. ^{1}H NMR of *mini*-PEG thiol in $CDCl_3$.

S4 Experimental procedures and results

S4.1 IBX-mediated oxidative *O*-demethylation of *mini*-lignin

Mini-lignin (100 mg, 0.26 mmol, 1 eq.) was dissolved in an aqueous methanol solution (5 mL, 1/1, v/v) before adding IBX (329 mg, 1.18 mmol, 4.5 eq.). The reaction mixture instantly turned dark brown. After stirring the mixture for 15 min at room temperature the mixture was precipitated into ultra-pure water. After centrifugation a brown solid was obtained, which was washed with an aqueous methanol solution (10%). Freeze drying resulted in brown powder (100 mg, 115%).

The yield of more than 100% is due to contamination by different iodobenzoic acid species. This is also evident from the ^{1}H NMR spectrum, which shows the characteristic chemical shifts for IBX and iodobenzoic acid in the range of 7.73 to 7.21 ppm (Figure S8). In addition, a broadening of the ^{1}H NMR signals indicated the presence of higher molecular weight fractions. This was confirmed by THF-GPC analysis (Figure S9). Further analysis by FTIR showed that no quinone signal was detected at 1660 cm^{-1}.

^{1}H NMR (500 MHz, DMSO-d_6) δ [ppm] = 8.51 – 7.82 (m, 1H), 7.78 – 7.14 (m, 1H), 7.05 – 5.73 (m, 2H), 4.32 – 3.30 (m, 3H).

^{13}C NMR (126 MHz, DMSO) δ [ppm] = 168.6, 141.0, 135.7, 132.9, 131.6, 130.8, 128.6, 121.7, 94.6, 49.1, 48.9, 30.6, 29.5, 17.7.

GPC (THF) $M_{n,\,app.}$ = 900 g·mol^{-1}, $M_{w,\,app.}$ = 1000 g·mol^{-1}.

FTIR (ATR): $\tilde{\nu}$ [cm^{-1}] = 3350 (s), 2335 (w), 1652 (s), 1601 (s), 1508 (m), 1436 (w), 1373 (w), 1340 (w), 1271 (m), 1224 (m), 1110 (w), 1016 (m), 827 (m), 740 (m), 690 (w), 580 (m), 515 (m), 474 (s).

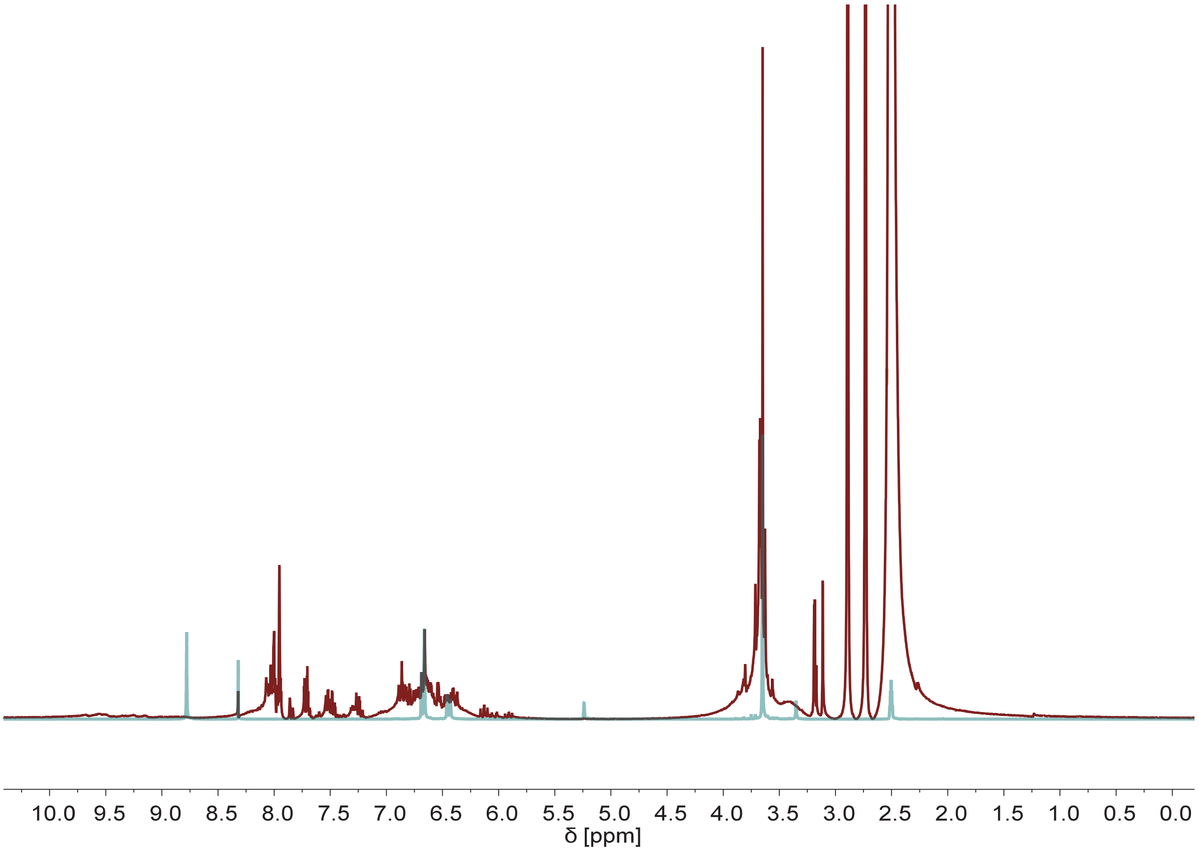

Figure S8. ^{1}H NMR of *mini*-lignin (blue) and *mini*-lignin after IBX-mediated oxidative *O*-demethylation (red) in DMSO-d_6.

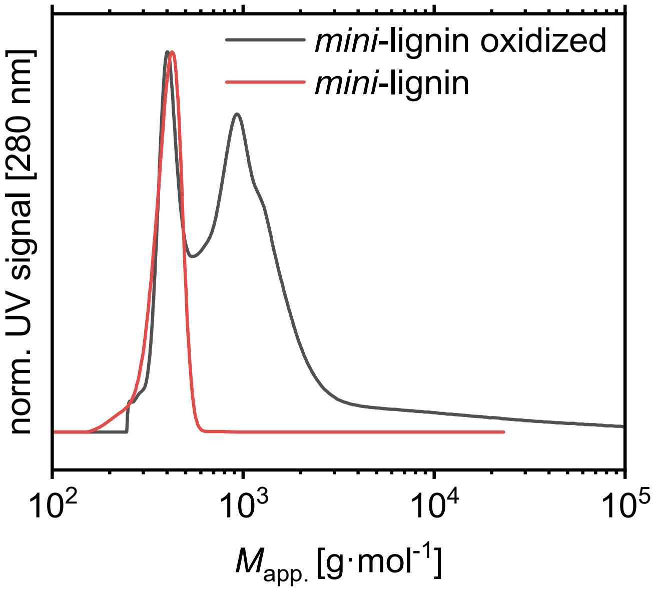

Figure S9. THF-GPC of *mini*-lignin before (red) and after (black) oxidation with IBX in MeOH.

S4.2 Oxidation of demethylated *mini*-lignin

The demethylated *mini*-lignin obtained was further reacted with various oxidizing agents. IBX, sodium periodate ($NaIO_4$) and ceric ammonium nitrate (CAN) were used.
Demethylated *mini*-lignin (100 mg, 0.29 mmol, 1 eq.) was dissolved in NMP (2.5 mL) and mixed with the oxidizing agent (4.5 eq., Table S1). The reaction mixture was stirred at room temperature for 15 min before it was precipitated into an aqueous methanol solution (1/10, v/v). After centrifugation and decanting of the supernatant, the samples were freeze-dried. All samples were in the form of brown powder.

Table S1. Details of the mass *m* and amount of substance *n* of the oxidizing agents used with the yield obtained.

	m [mg]	*n* [mmol]	Yield [mg]
IBX	365	1.31	65
$NaIO_4$	278	1.31	43
CAN	715	1.31	39

In contrast to the IBX-assisted *O*-demethylation of *mini*-lignin the demethylated *mini*-lignin appeared to be even more susceptible to cross-linking. For example, none of the derivatives were soluble in THF, so that only ^{1}H NMR analyses could be performed (Figure S10). As before, all the signals are highly broadened, suggesting a network in addition to the insolubility aspect.

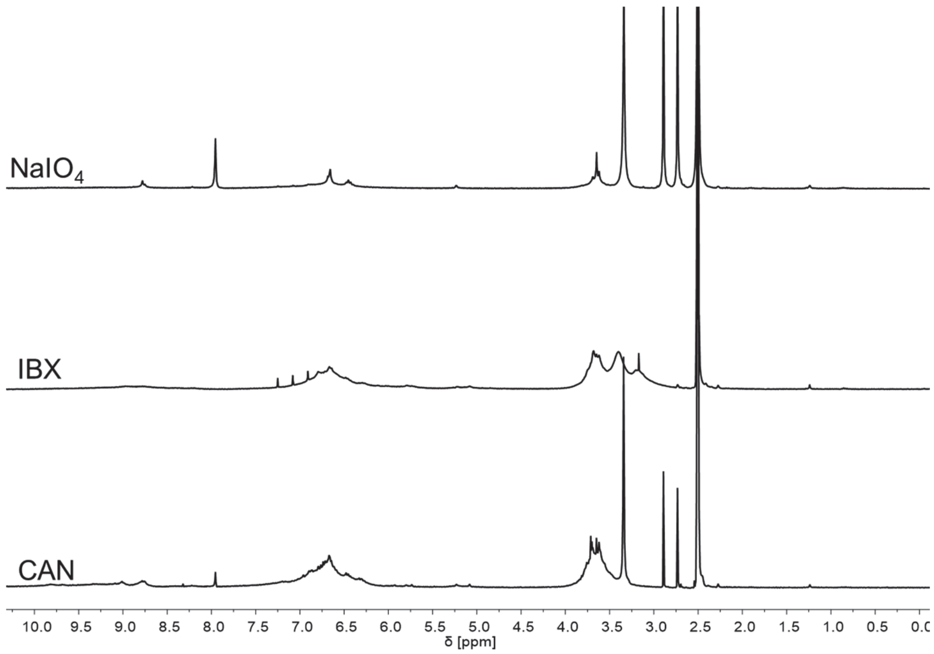

Figure S10. ^{1}H NMR in DMSO-d_6 of demethylated *mini*-lignin after oxidation in NMP with mentioned oxidizing agents and precipitation.

It seems reasonable to suggest that the proton in the benzylic position of the demethylated *mini*-lignin may present a challenge. As has been observed in previous work with different bisphenol derivatives, having protons in benzylic positions,[5] as well as in the field of triphenylmethane dyes,[10] it seems that the central proton can be abstracted, which may result in the formation of a conjugated π-system (Figure S11). It seems that this occurs as a result of oxidation. The formation of an *ortho*-quinone leads to the formation of an electron-withdrawing group, which causes the central proton to become acidic. If the hypervalent iodine species now attempts to oxidize a second aromatic ring during a second oxidation (Figure S11 I2 → I3), it appears that the abstraction of the α-CH hydrogen is favored. The I3 formed is then probably in an unstable intermediate state, which could result in an oxidative polycondensation.

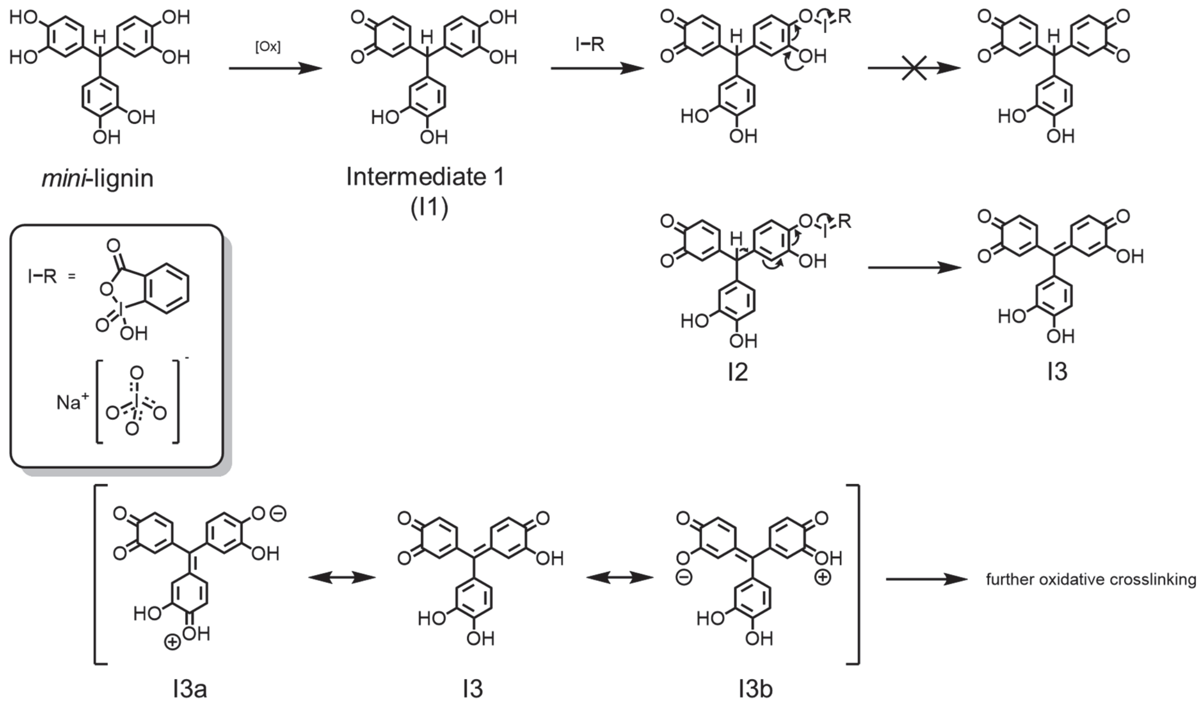

Figure S11. Presumed reaction scheme suggesting the oxidative cross-linking of demethylated *mini*-lignin.

S4.3 Thermal stability testing of TQ

Thermal stability was evaluated *via* thermogravimetric analysis (TGA) under inert atmosphere conditions in an argon stream and in tempered ovens under atmospheric conditions. TGA revealed that the 5% degradation temperature ($T_{5\%}$) is 330 °C, with the maximal degradation rate (T_{max}) occurring at 420 °C (Figure S12). Overall, no significant degradation was observed up to 190 °C ($T_{1\%}$), indicating that the material is stable at temperatures below 190 °C.

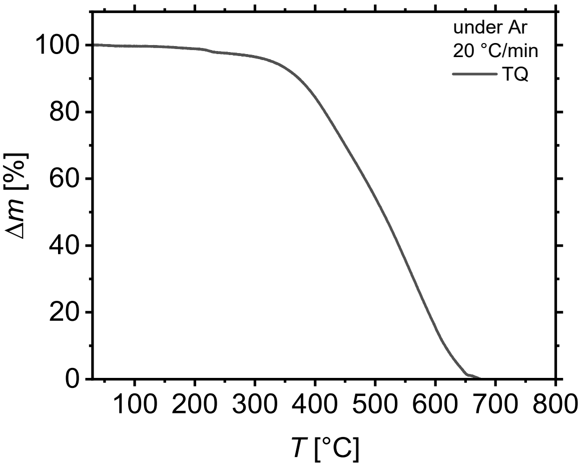

Figure S12. TGA analysis of TQ under argon with a heating rate of 20 °C·min^{-1}.

The storage of TQ batches (100 mg) in tempered ovens for 48 hours and subsequent analysis *via* ^{1}H NMR revealed that TQ is stable under these conditions until 120 °C (Figure S13A). At temperatures of 150 °C, new signals emerge in the ^{1}H NMR spectrum, indicating a change in the molecular structure. These changes may be attributed to quinone-quinone crosslinking.[11] At a temperature of 180 °C, the product formed upon storage of TQ becomes black and insoluble (Figure S13B). Consequently, no signals were observed in the ^{1}H NMR spectrum except DMSO-d_6 and water, which can be attributed to carbonization.

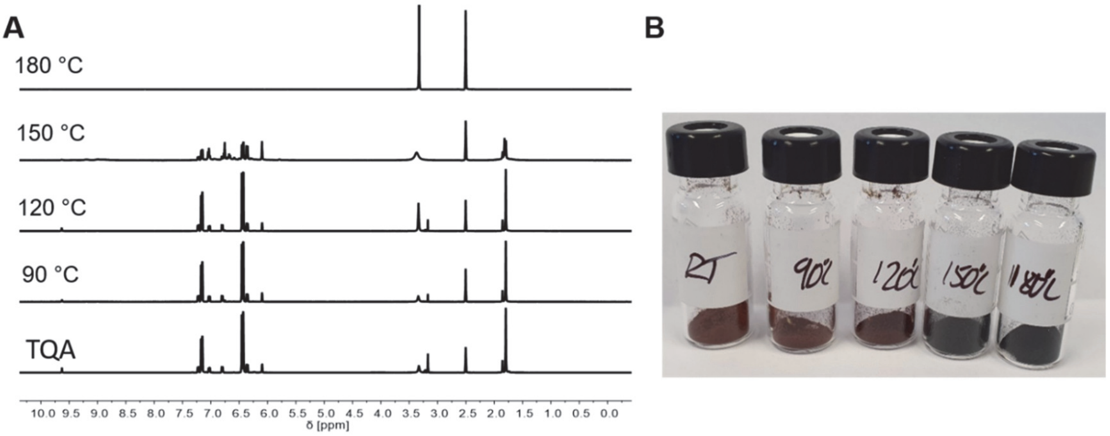

Figure S13. ^{1}H NMR in DMSO-d_6 of TQ after storage at different temperatures for 48 h (A). Optical tracing of TQ after storage at different temperatures (B).

S4.4 Determining the reactivity in solution

To a solution of TQ (58 mg, quinone groups: 0.47 mmol, 1 eq.) in NMP (5.8 ml) ethane thiol (87 mg, 1.41 mmol, 3 eq.) was added. The reaction mixture was stirred at room temperature. After 15 min a sample was taken and measured *via* UHPLC analysis (10 µL diluted into 1 mL aqueous acetonitrile (80%) and UV/vis spectroscopy (10 µL diluted into 1 mL NMP).

The formation of different *Michael* adducts could be observed *via* UHPLC analysis (FigureS14A). All *ortho*-quinone species reacted with ethane thiol (cf. grey and red). Here the three-fold thiol adduct represents the main product (72%), whereas twice (22%) and four-fold (6%) thiol products are formed as well. Additionally, the UV/vis data clearly shows the vanishing of the *ortho*-quinone band at 380 nm due to the reaction with ethane thiol (FigureS14B).

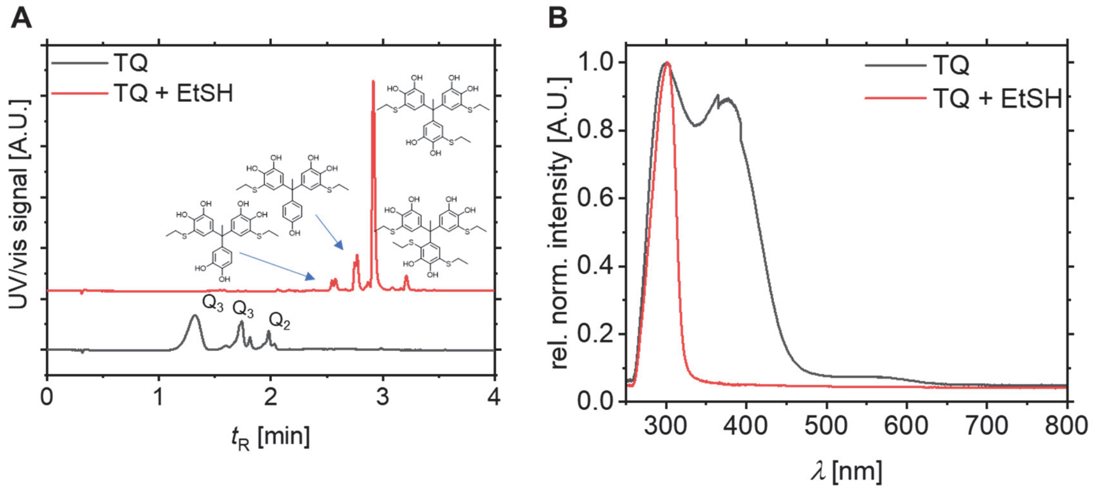

Figure S14. Reaction monitoring *via* UHPLC (A) and UV/vis spectroscopy (B) of TQ with ethane thiol in NMP at room temperature after 15 min.

Further characterization was performed *via* NMR and FTIR, proving the successful conversion of TQ with ethane thiol.

^{1}H NMR (500 MHz, DMSO-*d*6) δ [ppm] = 9.18 (s, 2H), 8.43 (s, 2H), 6.56 (dd, *J* = 15.5, 8.5 Hz, 1H), 6.44 – 6.30 (m, 2H), 2.68 (p, *J* = 7.3 Hz, 2H), 2.55 – 2.42 (m, 1H), 2.22 (s, 1H), 2.08 (s, 3H), 1.93 (d, *J* = 9.0 Hz, 1H), 1.10 (t, *J* = 7.3 Hz, 3H), 0.90 (t, *J* = 7.4 Hz, 2H).
^{13}C NMR (126 MHz, DMSO) δ [ppm] = 206.6, 147.8, 144.2, 143.5, 141.9, 140.6, 140.3, 129.3, 121.3, 114.4, 54.5, 51.9, 50.4, 30.3, 27.7, 25.8, 14.3.

UHPLC-ESI-MS (λ = 280 nm, gradient 10-90% B with A: H_2O + 0.1% FA and B: ACN + 0.1% FA);

t_R = 2.57 min, found (m/z): 473.21 $[M-H]^-$, calculated (m/z): 473.10 $[M-H]^-$;

t_R = 2.77 min, found (m/z): 457.27 $[M-H]^-$, calculated (m/z): 457.12 $[M-H]^-$;

t_R = 2.57 min, found (m/z): 533.56 $[M-H]^-$, calculated (m/z): 533.11 $[M-H]^-$;

t_R = 2.57 min, found (m/z): 592.42 $[M-H]^-$, calculated (m/z): 592.12 $[M-H]^-$.

FTIR (ATR): $\tilde{\nu}$ [cm^{-1}] = 3375 (s), 2975 (m), 2923 (m), 2867 (w), 1695 (m), 1593 (m), 1473 (s), 1411 (s), 1332 (m), 1261 (s), 1215 (s), 1172 (s), 1124 (m), 1056 (w), 958 (m), 883 (w), 817 (w), 732 (w), 540 (w).

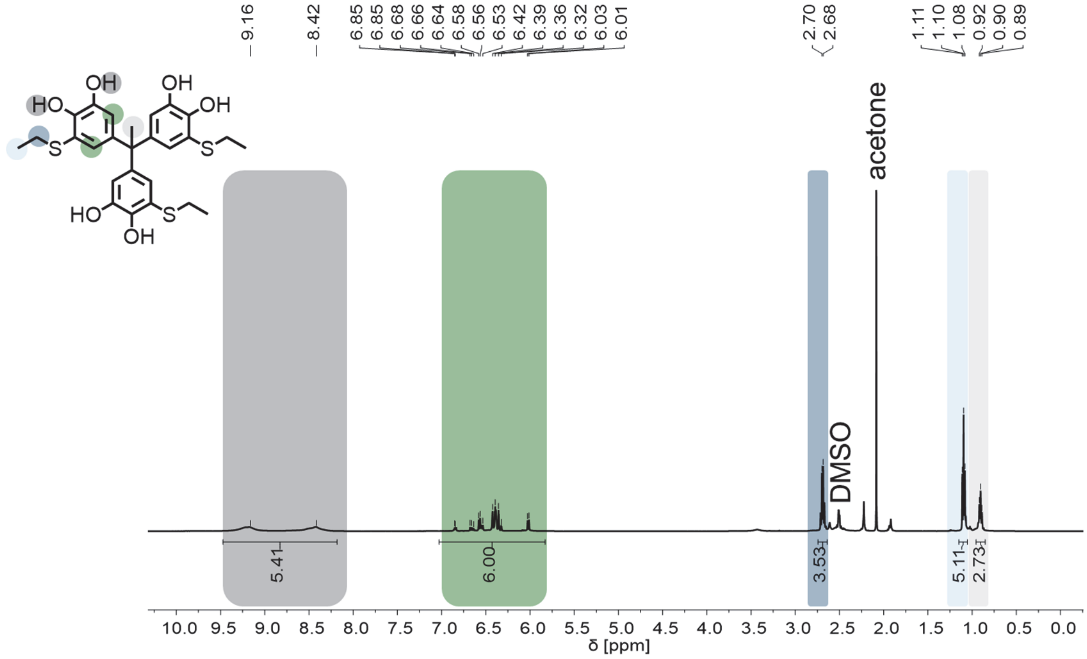

Figure S15. ^{1}H NMR spectra of TQ-$(EtSH)_3$ in DMSO-d_6.

S4.5 Hydrolysis of 2K System

To prove TCC formation, a two-component (2K) adhesive system was cured under standard conditions before it was hydrolyzed. Therefore, TQ/TTMP (1/1) was cured at 120°C for 17 h. The adhesive was then pulverized, and 300 mg of the adhesive powder was added to a hydrochloric acid solution (15 mL, 5 M). The mixture was heated at 70 °C for 5 h. The organic residues were then extracted from the aqueous solution with ethyl acetate (3x 15 mL). The combined organic phases were dried over magnesium sulfate and under reduced pressure.

The resulting product could be analyzed by UHPLC to demonstrate the formation of TCCs by showing *Michael* adducts of TQ with mercaptopropionic acid (Figure S16).

UHPLC-ESI-MS (λ = 280 nm, gradient 10-90% B with A: H_2O + 0.1% FA and B: ACN + 0.1% FA);
t_R = 1.48 min, found (m/z): 545.13 $[M-H]^-$, calculated (m/z): 545.09 $[M-H]^-$;
t_R = 1.75 min, found (m/z): 457.18 $[M-H]^-$, calculated (m/z): 457.10 $[M-H]^-$;
t_R = 1.87 min, found (m/z): 561.27 $[M-H]^-$, calculated (m/z): 561.08 $[M-H]^-$;
t_R = 2.04 min, found (m/z): 665.18 $[M-H]^-$, calculated (m/z): 665.08 $[M-H]^-$;
t_R = 2.48 min, found (m/z): 769.13 $[M-H]^-$, calculated (m/z): 769.84 $[M-H]^-$.

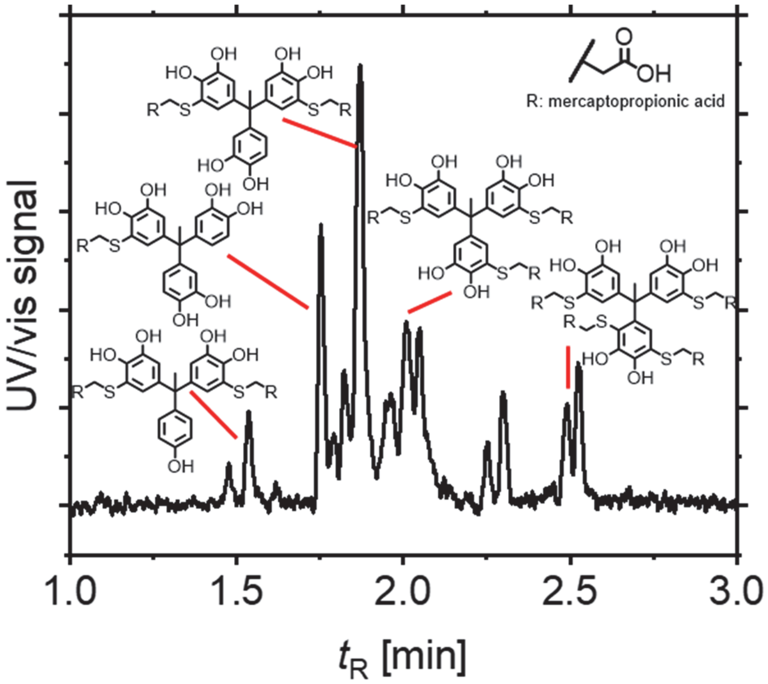

Figure S16. UHPLC chromatogram of the hydrolyzed 2K adhesive from TQ/TTMP (1/1).

S4.6 Preparation lap shear testing with debonding

S4.6.1 Lap shear testing

All specimens were cleaned with isopropanol and acetone in advance.

The 2K adhesive systems were prepared by adding TQ and tri-thiols to a 10 mL polypropylene container in the corresponding ratio. The container was then placed in a mixer for bladeless shear mixing (SpeedMixer) and the compounds were blended using a mixing ramp for 2 min and 40 s at maximum 3500 rpm, resulting in a homogeneous viscous paste.

The 2K adhesive was applied to an aluminum substrate with 20 mg per 140 mm^2 of surface area. A second substrate was then added on top of the applied adhesive, creating an overlap between the two substrates. Both substrates were fixed together, and the expelled glue was removed prior to curing in an oven at constant temperatures for 17 h. Afterwards, the samples were equilibrated at room temperature for 2 h.

The adhesive strength was determined using a lap shear test conducted at a testing speed of 0.05 $mm \cdot s^{-1}$. It was calculated by dividing the maximum force recorded during the test by the overlap area of the bonded joint. Only the actual adhesive contact area was considered for this calculation. Adhesive strength was reported in units of $N \cdot mm^{-2}$ (equivalent to MPa), representing the maximum applied force divided by the bonded area.

S4.6.2 Debonding procedure

Prior to electrochemical debonding tests, the systems were tested for resistance and electrical conductivity. For this purpose, a multimeter was applied to the corresponding test systems. The composite system was then connected to the laboratory star. The maximum current was limited to 1 A for safety reasons. A voltage was then applied for the specified duration, usually 30 minutes.

To determine the time-dependent current or voltage curve, the tests were filmed and the values shown on the display were noted every 10 seconds.

S4.6.3 Solvent robustness testing procedure

The bonded systems were allowed to cool to room temperature after being cured between the test specimens according to the described procedure, before being immersed in the specified solvent in a beaker for 2 hours. Afterwards, they were removed from the solvent and dried with a cloth. For each solvent, three composite systems were tested using the described lap shear method.

S4.7 Statistical Analysis

Depending on the specific investigation, between two and ten bonded specimens were prepared for each test series, and their adhesive strengths were determined using lap shear tests. Based on the individual measured values, the mean and standard deviation were calculated. Results are reported as the mean value together with the range of one standard deviation (mean ± SD). To assess the significance of differences between various measurements, statistical significance was determined using two-sample t-tests. Significance levels are indicated as follows: $p < 0.05$ (*), $p < 0.01$ (**), $p < 0.001$ (***). All statistical analyses were performed using OriginPro 2022 (Version 9.9.0.225).

S5 Experimental Data

S5.1 Characterization of the thiols

Table S2. Molecular weight, thiol density and viscosity of the thiols used.

	Molecular weight ($g \cdot mol^{-1}$)	Thiol density ($mmol \cdot g^{-1}$)	Viscosity (mPa·s)
mini-PEG-SH	182	5.55	5
TTMP	398	7.53	164
$ETTMP_{700}$	700	4.29	212
$ETTMP_{1300}$	1300	2.31	421

S5.2 Temperature-dependent oscillatory tests *via* rheology

The curing behavior of different 2K mixtures with a quinone/thiol ratio (Q/T) of 1/1 was investigated *via* rheological analysis, employing oscillatory tests with a constant amplitude (0.1%) and a constant frequency (1 Hz). The storage (G') and loss (G'') modulus of the non-cured mixtures changed with temperature, transitioning from a viscous to a solid state (Figure S17). The sol-/gel-transition temperatures (T_{sg}) varied: TQ/TTMP at 11.5 °C, TQ/$ETTMP_{1300}$ at 26.0 °C, and TQ/$ETTMP_{700}$ at 9.1 °C.

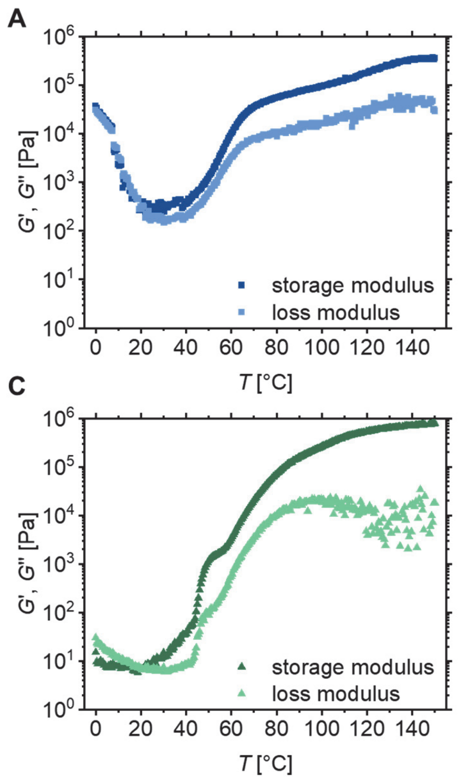

Figure S17. Temperature-dependent functions of storage (*G*') and loss (*G*'') modulus determined with oscillatory tests *via* rheology at a with a constant amplitude (0.1%) and a constant frequency (1 Hz). TQ/TTMP (1/1) (A), TQ/ETTMP$_{700}$ (1/1) (B) and TQ/ETTMP$_{1300}$ (1/1) (C).

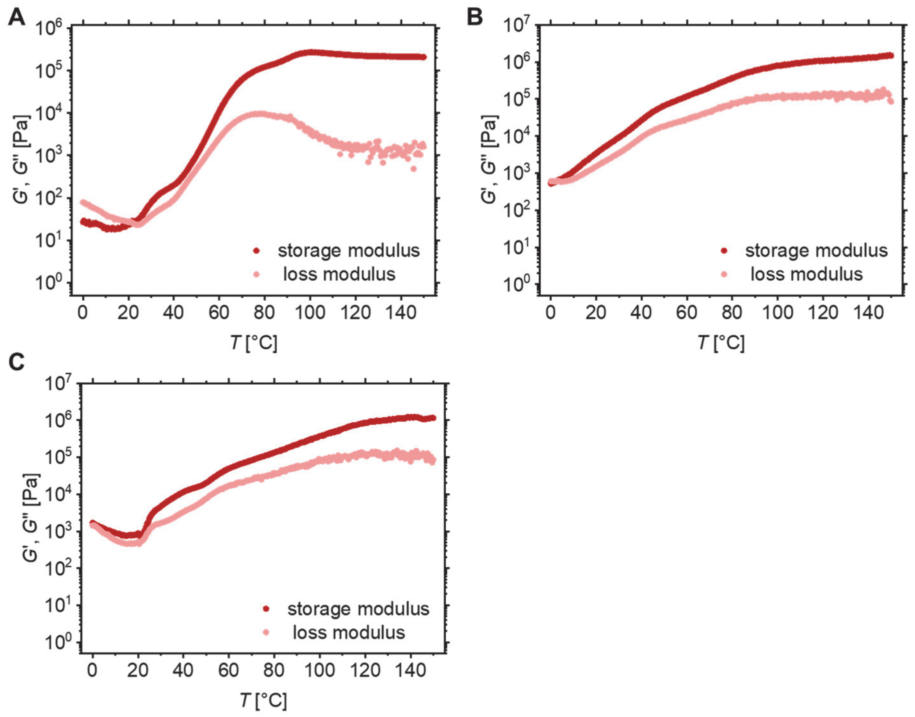

Figure S18. Temperature-dependent functions of storage (*G*') and loss (*G*'') modulus determined with oscillatory tests *via* rheology at a with a constant amplitude (0.1%) and a constant frequency (1 Hz). TQ/ETTMP$_{700}$ (1/0.8) (A), TQ/ETTMP$_{700}$ (1/0.8) +5 wt% CB (B) and TQ/ETTMP$_{700}$ (1/0.8) +5 wt% CB +5 wt% IL (C).

S5.3 Isothermal experiments rheology

Isothermal experiments showed that higher curing temperatures resulted in increased final strength and faster curing, considering the complex viscosity (η^*) over time (Figure S19). The mixtures exhibited a pot-life of at least 20 minutes at 25 °C. Additionally, after storage at room temperature for three days, η^* increased by two orders of magnitude, but the adhesive mixture remained smearable.

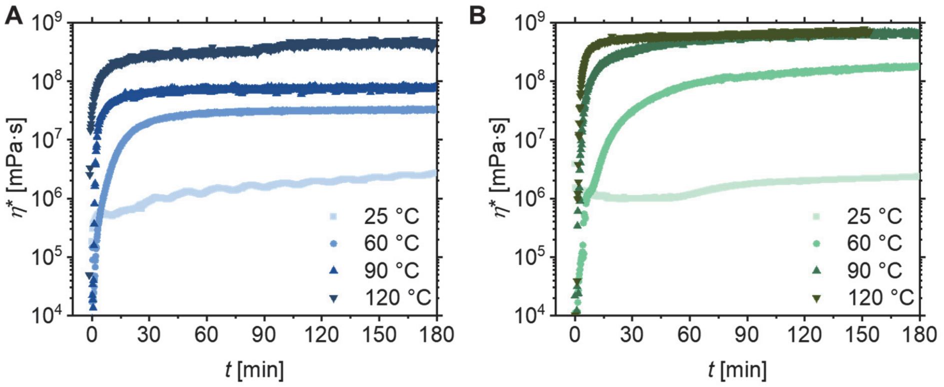

Figure S19. Time-dependent function of complex viscosity (η^*) determined with oscillatory tests at isothermal conditions of TQ/TTMP (1/1) (A) and TQ/ETTMP$_{1300}$ (1/1) (B).

S5.4 FTIR analysis

FTIR is an effective analytical method for monitoring the conversion of catechols to *ortho*-quinones. Of particular interest are the hydroxyl vibrational bands around 3300 cm^{-1} and the conjugated carbonyl vibrational band at 1660 cm^{-1}.

S5.4.1 Transition from THPE to TQ

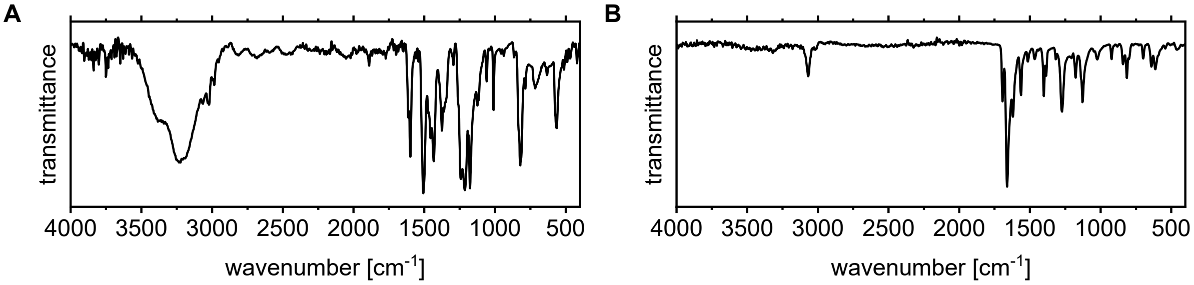

Figure S20. FTIR (ATR) analysis of THPE (A) and TQ (B).

S5.4.2 Curing behavior of TQ with *mini*-PEG-SH with different curing conditions

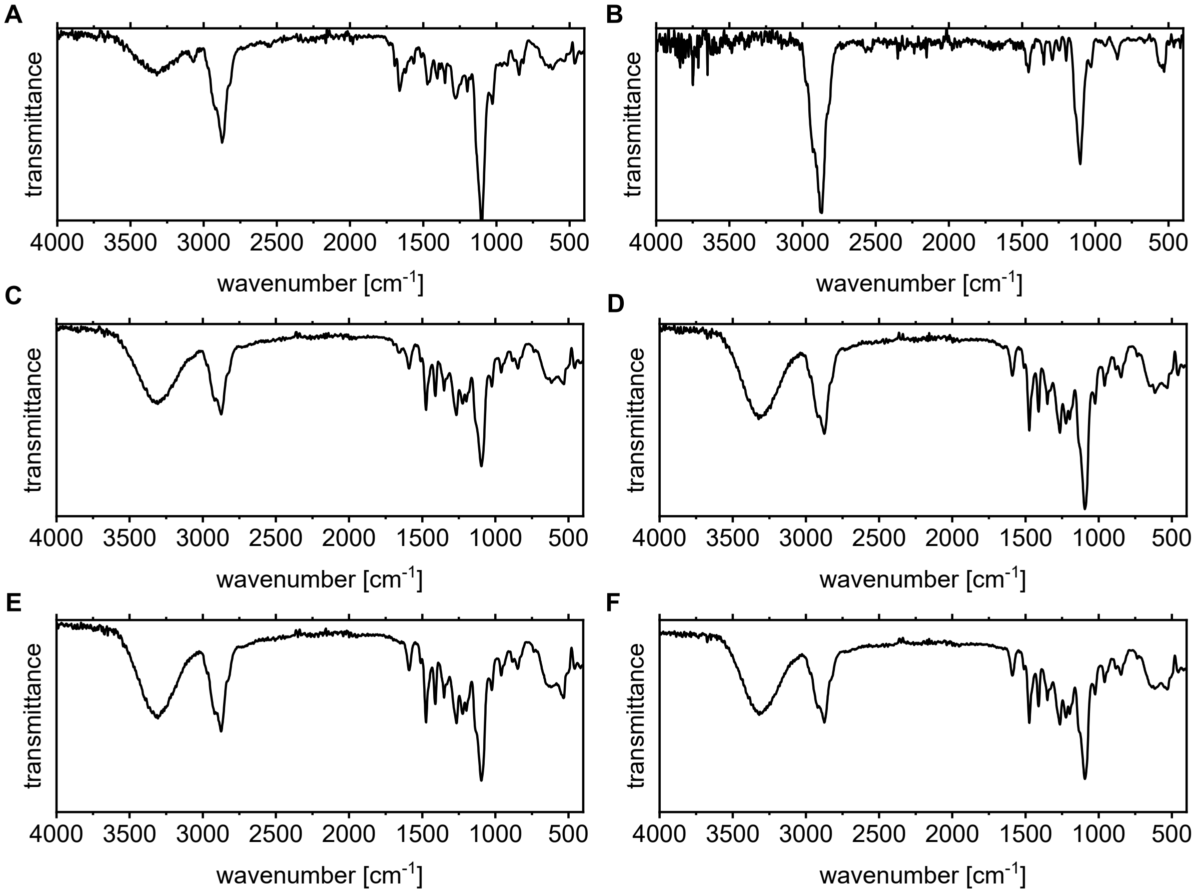

Figure S21. FTIR (ATR) analysis of *mini*-PEG-SH (A) and mixtures of TQ/mini-PEG-SH (Q/T, 1/1) freshly mixed (B) and after storing it at temperatures of RT (C), 60 °C (D)*, 90 °C (E) and 120 °C (F) for 17 h. (* Mixture at 60°C cured for 36 h).

S5.4.3 TQ + trithiols (Q/T, 1/1)

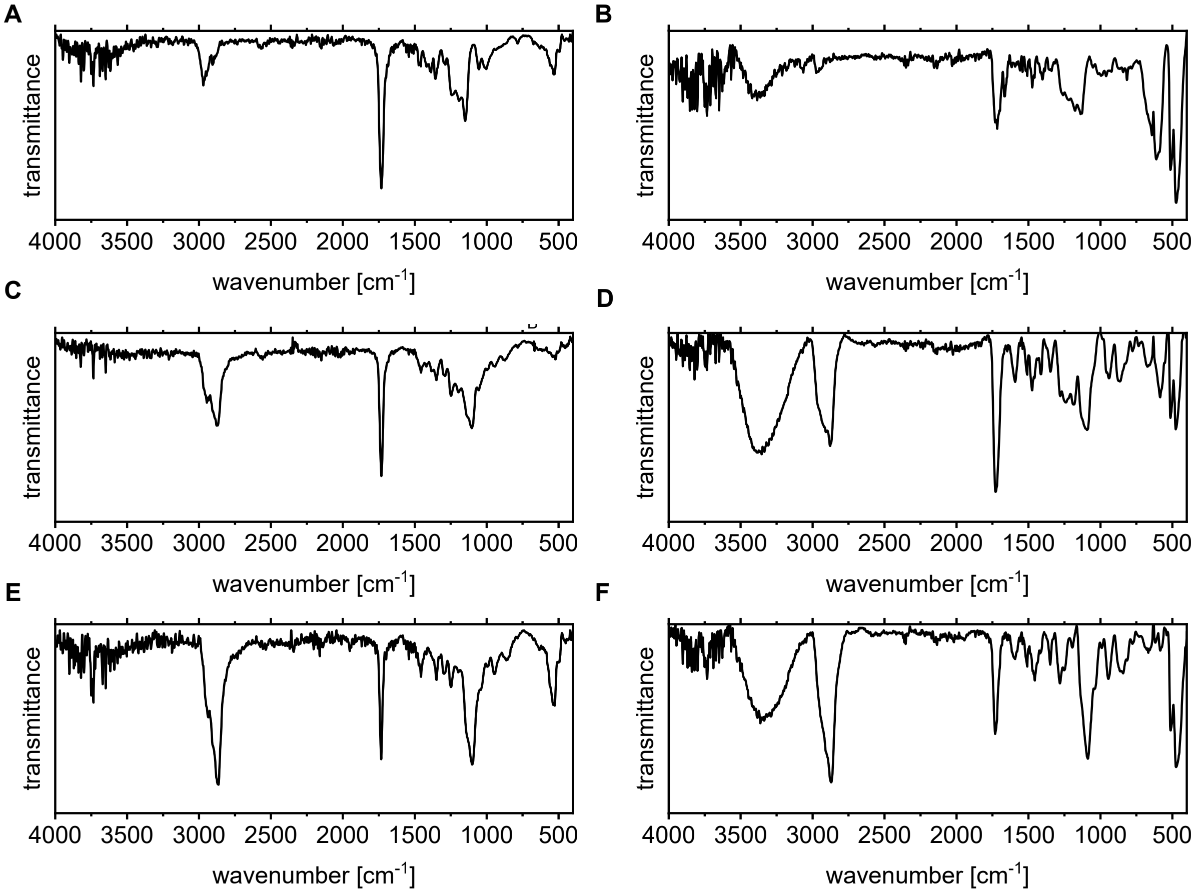

Figure S22. FTIR (ATR) analysis of TTMP (A), ETTMP$_{700}$ (C), ETTMP$_{1300}$ (E) and mixtures of TQ with TTMP (B), ETTMP$_{700}$ (D), ETTMP$_{1300}$ (F) (Q/T, 1/1) after curing at 120 °C for 17 h.

S5.4.4 TQ + $ETTMP_{700}$ (Q/T, 1/0.8)

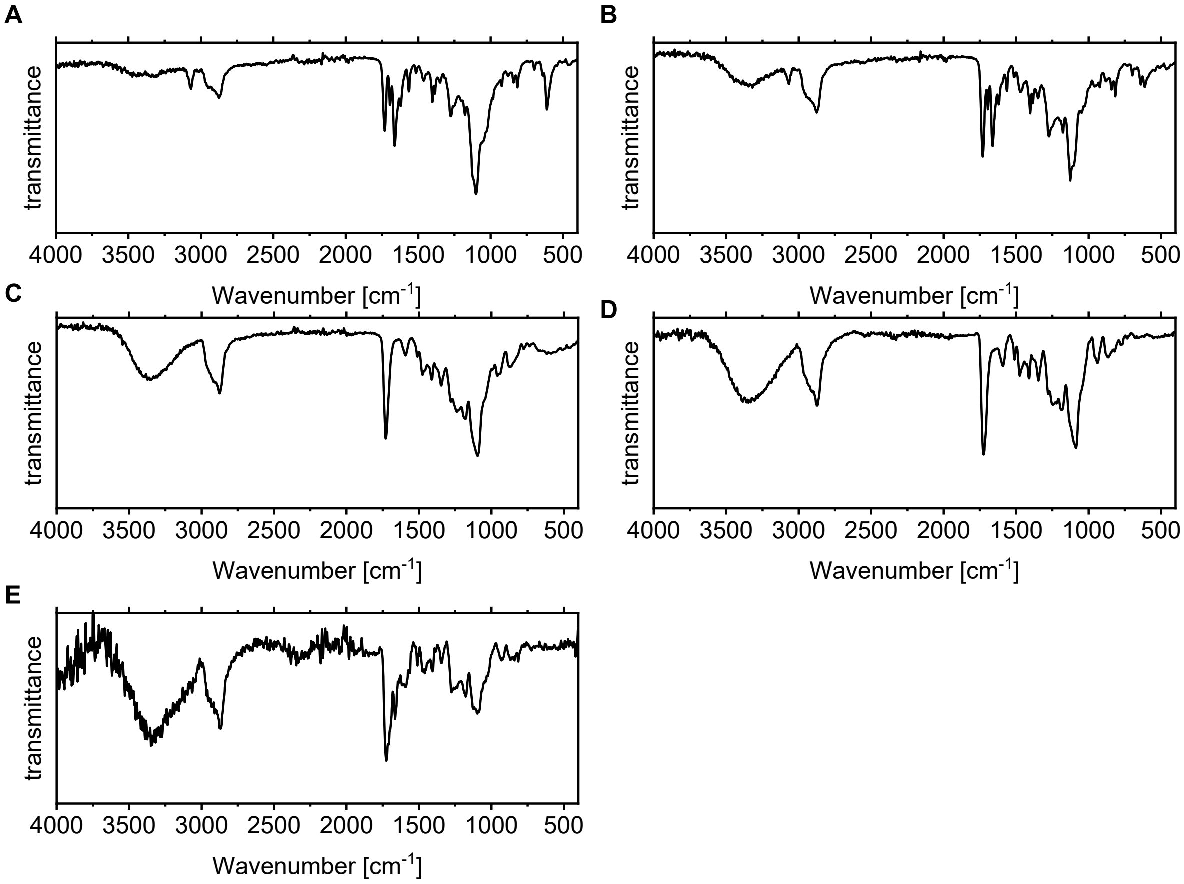

Figure S23. FTIR (ATR) analysis of TQ/$ETTMP_{700}$ (1/0.8) freshly mixed (A), stored at RT for 3 days (B) and after curing it at 120°C for 17 h (C). Additionally, the CB-filled (D) and the CB- and IL-filled (E) TQ/$ETTMP_{700}$ (1/0.8) systems are displayed, each containing a 5 wt% additive.

S5.4.5 Analysis after Debonding experiment

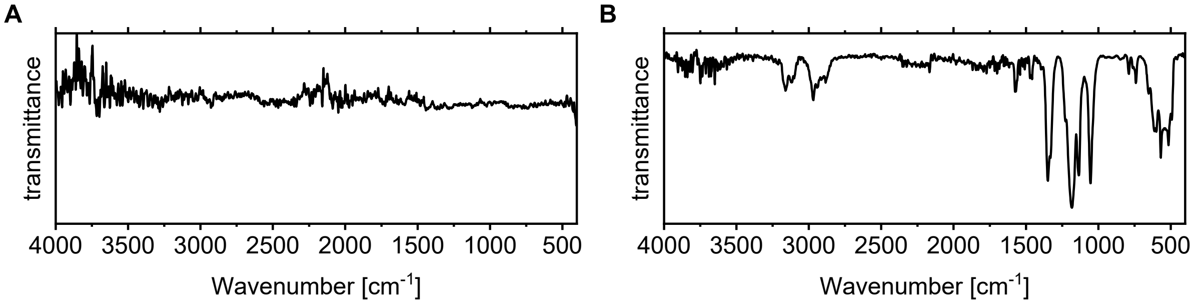

Figure S24. FTIR (ATR) analysis of carbon black (A) and BMIM·NTf_2 (B).

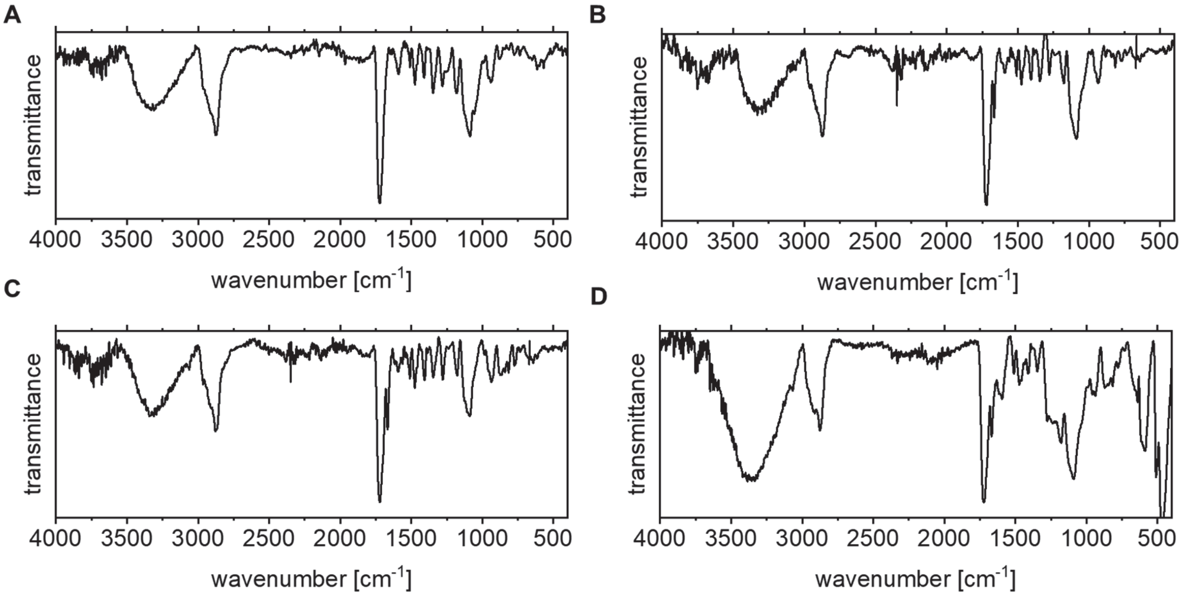

Figure S25. FTIR (ATR) analysis of TQ/$ETTMP_{700}$ (Q/T, 1/1.1) with 5 wt% CB and 5wt% BMIM·NTf_2 cured for 17 h at 120 °C (A) and after voltage application (*E* = 9V) for 5 min (B), 10 min (C) and 30 min (D).

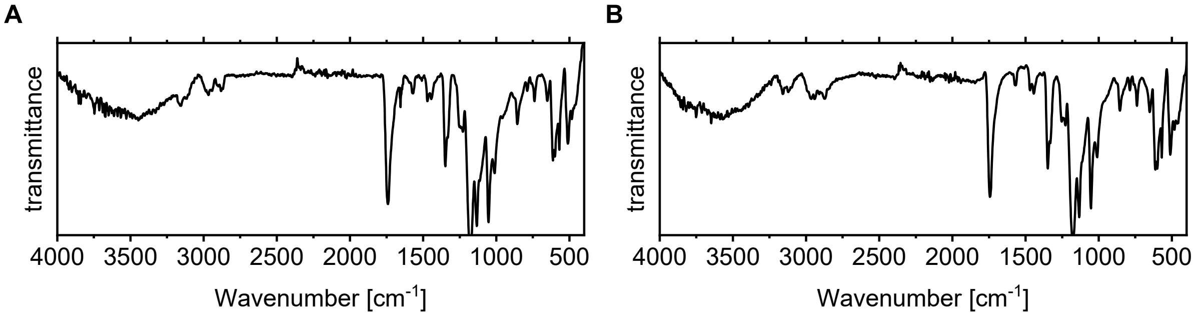

Figure S26. FTIR (ATR) analysis of TQ/$ETTMP_{700}$ (Q/T, 1/1.1) with 5 wt% CB and 5wt% BMIM·NTf_2 cured for 17 h at 90 °C from the mini display studies. After voltage application (*E* = 9V) for 6.5 min (A) and after 4 days without voltage application (B).

S5.5 Thermogravimetric analysis (TGA)

Table S3. 5% degradation temperature ($T_{5\%}$) and maximal degradation temperature (T_{max}) of the different cured adhesive mixtures (Q/T, 1/1).

TQ/	$T_{5\%}$ [°C]	T_{max} [°C]
TTMP	305	344
$ETTMP_{700}$	324	353
$ETTMP_{1300}$	332	378

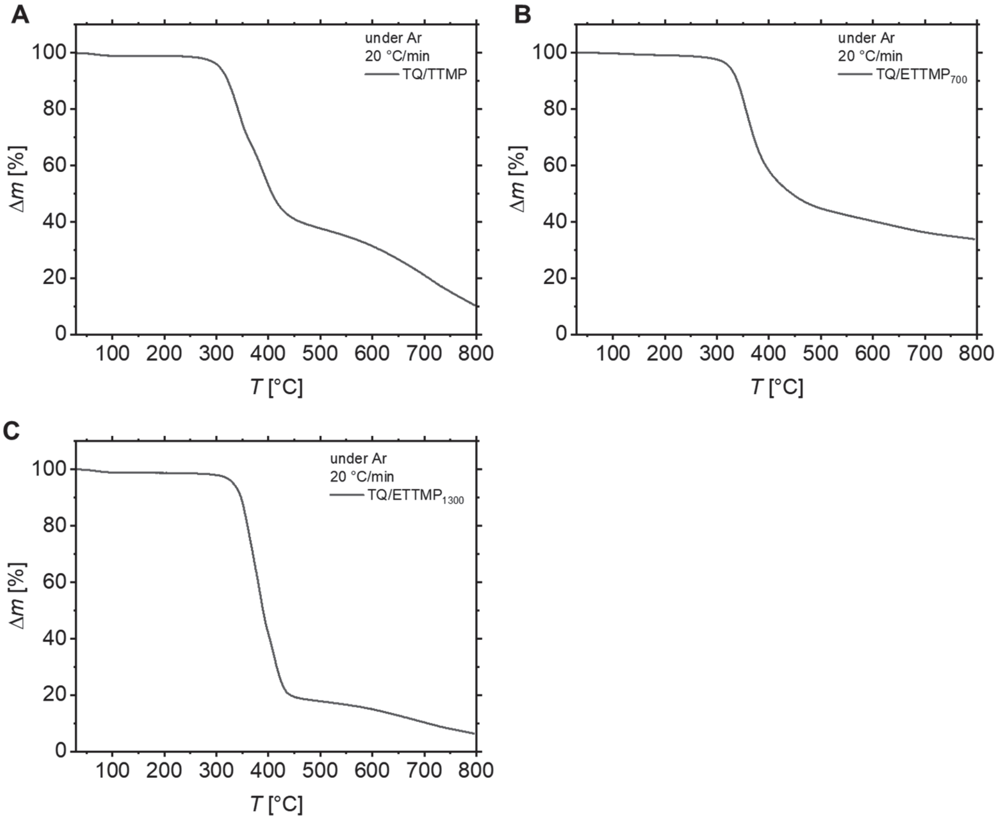

Figure S27. Thermogravimetric analyses of the cured 2K adhesive mixtures from TQ with TTMP (A), $ETTMP_{700}$ (B) and $ETTMP_{1300}$ (C) (Q/T, 1/1).

S5.6 Differential scanning calorimetry (DSC)

S5.6.1 DSC analysis of non-cured adhesive mixtures

Table S4. Determined onset temperatures (T_{onset}) of given non-cured 2K adhesive mixtures at a heating rate of 2 °C·min^{-1}.

	T_{onset} [°C]
TQ/TTMP (1/1)	30.6
TQ/ETTMP$_{700}$ (1/1)	29.6
TQ/ETTMP$_{1300}$ (1/1)	29.0
TQ/ETTMP$_{700}$ (1/0.8)	37.4
TQ/ETTMP$_{700}$ (1/0.8) + 5wt% CB	32.1
TQ/ETTMP$_{700}$ (1/0.8) + 5wt% CB + 5wt% IL	29.0

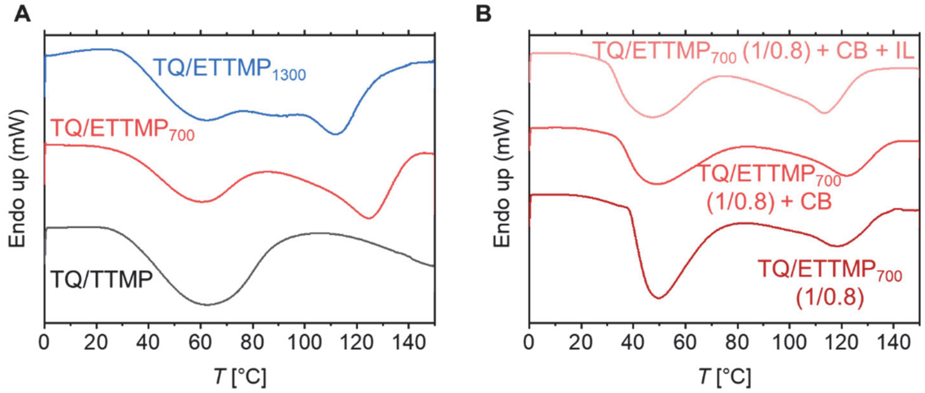

Figure S28. DSC analysis of non-cured 2K adhesive mixtures consisting of TQ with TTMP, ETTMP$_{700}$ and ETTMP$_{1300}$ (Q/T, 1/1) (A) and of non-cured TQ/ETTMP$_{700}$ (Q/T, 1/0.8) with CB and IL. The heating rate was 2 °C·min^{-1}.

S5.6.2 DSC analysis of cured adhesive mixtures

Table S5. Determined glass transition temperatures (T_g) of given cured 2K adhesive mixtures at a heating rate of 2 °C·min^{-1}.

	T_g [°C]
TQ/TTMP (1/1)	17.8
TQ/ETTMP$_{700}$ (1/1)	35.8
TQ/ETTMP$_{1300}$ (1/1)	-9.3
TQ/ETTMP$_{700}$ (1/0.8)	26.2
TQ/ETTMP$_{700}$ (1/0.8) + 5wt% CB	25.0
TQ/ETTMP$_{700}$ (1/0.8) + 5wt% CB + 5wt% IL	23.6

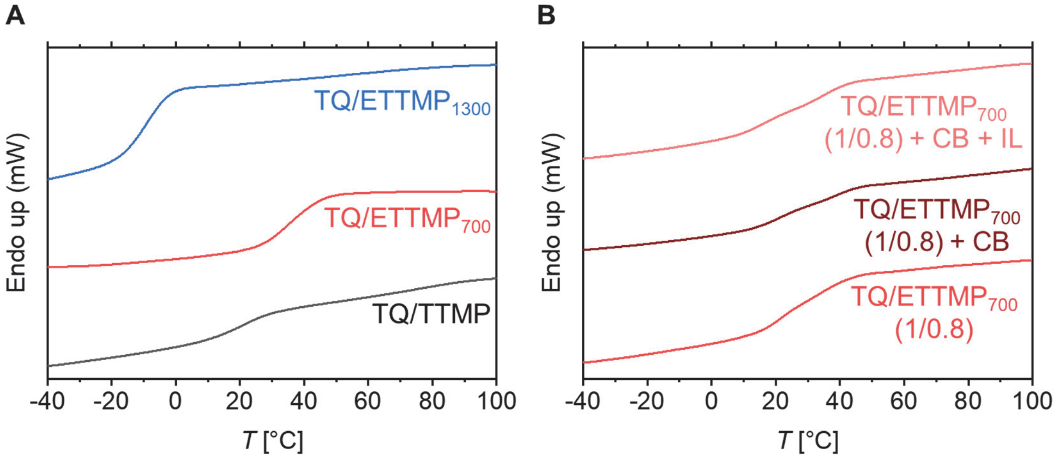

Figure S29. DSC analysis of cured 2K adhesive mixtures consisting of TQ with TTMP, $ETTMP_{700}$ and $ETTMP_{1300}$ (Q/T, 1/1) (A) and of cured 2K adhesive mixtures consisting of TQ and $ETTMP_{700}$ (Q/T, 1/0.8) with CB (5 wt%) and BMIM·NTf_2 (5 wt%) as ionic liquid (IL) (B). The second heating curve is shown with a heating rate of 10 °C·min^{-1}.

S5.7 Lap Shear Testing

S5.7.1 Temperature-dependent curing (60 °C/90 °C)

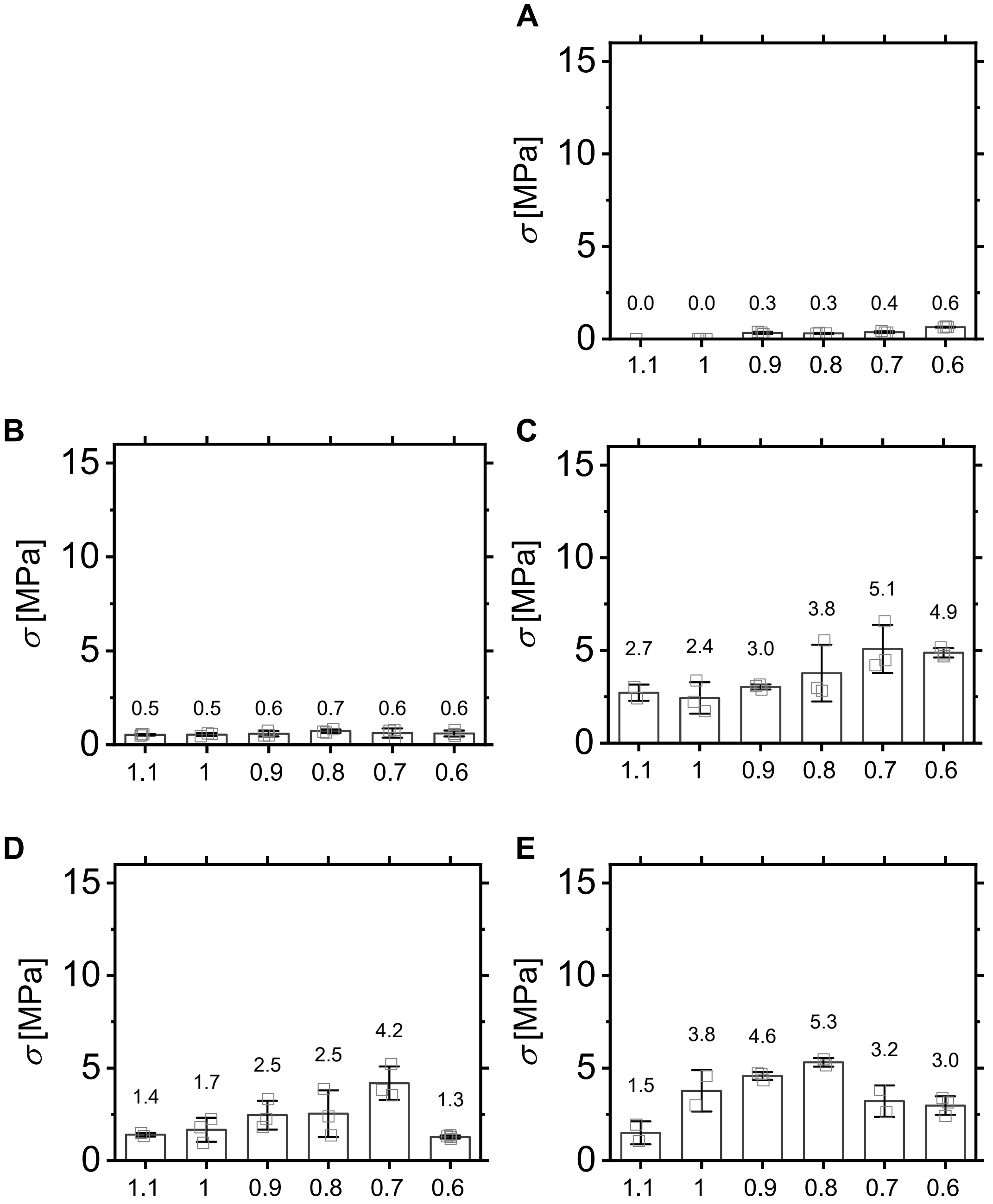

Figure S30. Shear strength including standard deviation determined from lap shear tests of TQ/thiol adhesives at different Q/T ratios, cured at 60 °C (left) and 90 °C (right) for 17 h. TTMP (A), $ETTMP_{700}$ (B, C) and $ETTMP_{1300}$ (D, E)

S5.7.2 TQ/ETTMP700 (1/0.8) + CB + IL (incl. debonding)

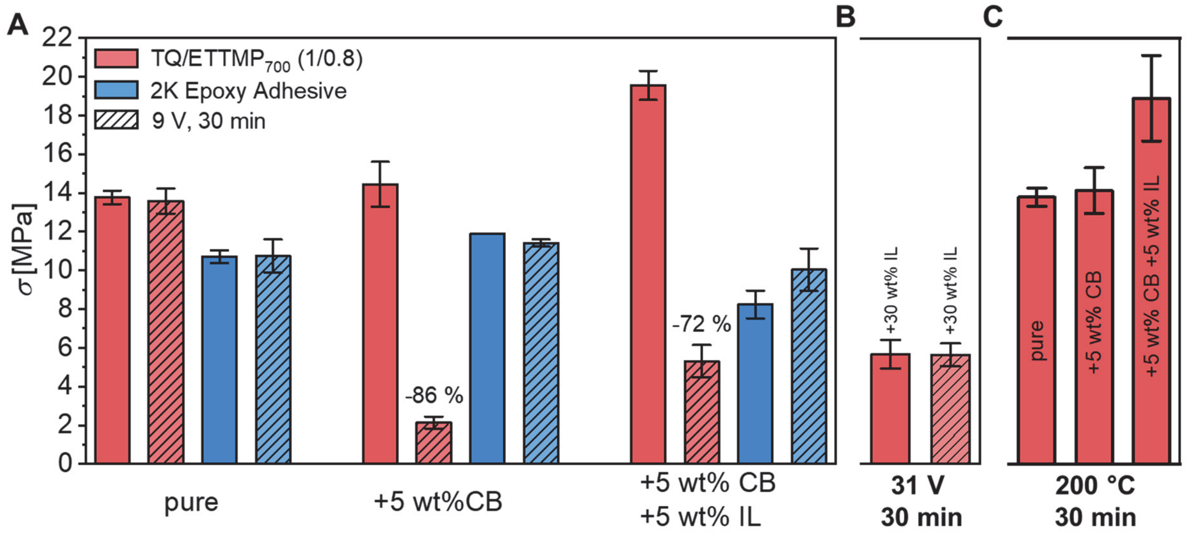

Figure S31. Shear strength including standard deviation determined from lap shear tests of TQ/ETTMP700 (Q/T, 1/0.8) and a commercially available 2K Epoxy adhesive without and with conductive additives such as carbon black (CB) and BMIM NTf2 as ionic liquid (IL) before and after voltage application (E = 9 V for 30 min) (A). Lap shear tests of TQ/ETTMP700 (Q/T, 1/0.8) with 30 wt% BMIM·NTf2 before and after voltage application (E = 31 V for 30 min) (B). TQ/ETTMP700 (Q/T, 1/0.8) without and with conductive additives such as CB and BMIM NTf2 after heating at 200 °C for 30 min (C). The TQ/ETTMP700 based adhesives were cured for 17 h at 120°C and the epoxy-based adhesives for 24 h at room temperature.

S5.7.3 TQ/ETTMP700 (1/1.1) + CB + IL (incl. debonding)

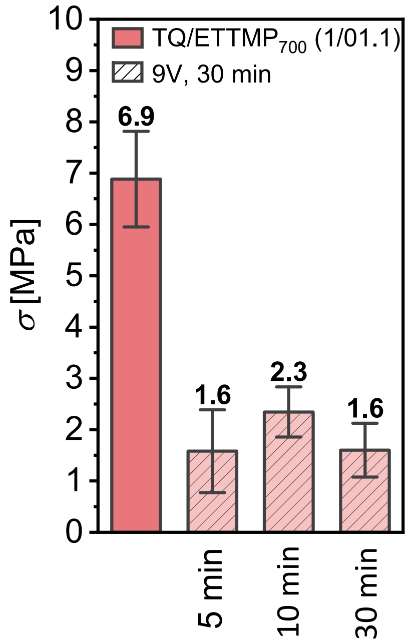

Figure S32. Shear strength including standard deviation determined from lap shear tests of TQ/ETTMP700 with conductive additives carbon black (CB) and BMIM·NTf2 as ionic liquid (IL) before and after voltage application (E = 9 V). The x axis indicates the duration of the voltage application. The TQ/ETTMP700 based adhesives were cured for 17 h at 120°C.

S5.7.4 Fracture pattern analysis

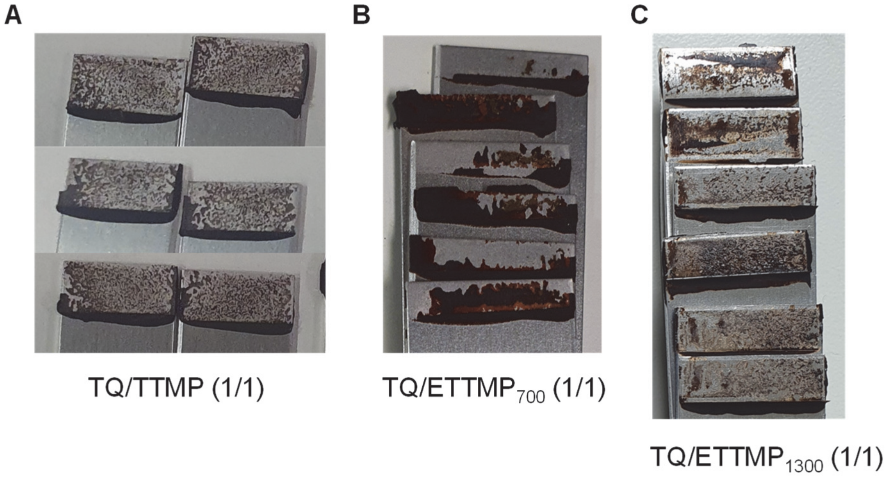

Figure S33. Tested bonded systems of TQ-based adhesives with TTMP (A), $ETTMP_{700}$ (B) and $ETTMP_{1300}$ (C) with a Q/T: 1/1 cured für 17 h at 120 °C.

S5.7.5 Substrate compatibility of IonoBlackTQ

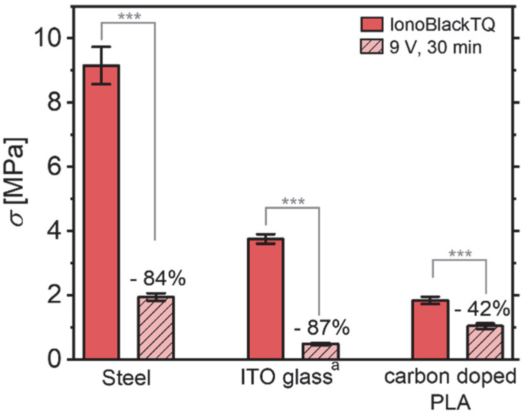

Figure S34. Bonding/debonding performance metrics of IonoBlackTQ across diverse substrates. Shear strength including standard deviation determined from lap shear tests before and after voltage application (E = 9 V, 30 min). The IonoBlackTQ adhesives systems were cured for 17 h at 120°C. [a]Substrate failure without debonding. Significance levels are indicated as follows: $p < 0.05$ (*), $p < 0.01$ (**), $p < 0.001$ (***).

S5.7.6 Solvent resistance testing

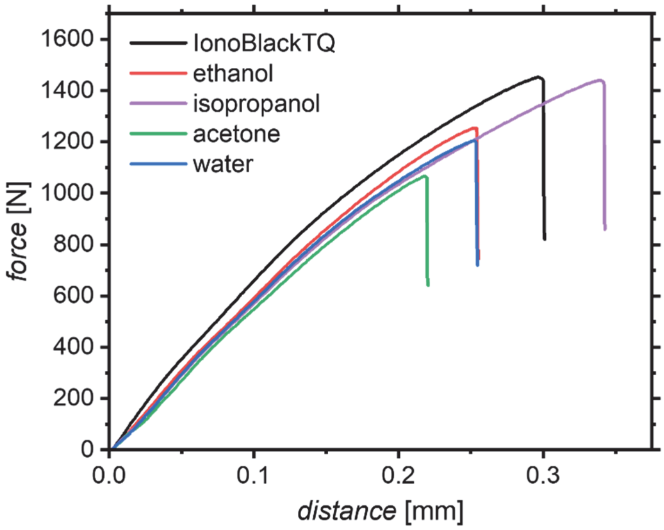

Figure S35. Representative force distance curves from lap shear tests of IonoBlackTQ before and after immersion in solvent baths (2 h at room temperature). The adhesive was cured for 17 h at 120 °C and equilibrated (24 h) prior to solvent exposure. The adhesive retained substantial bonding strength and failure remains abrupt as indicated by the sharp kink at maximum force.

S5.8 Thermographic analysis

The experiment was based on a passive thermographic measurement method to analyze the thermal response of adhesive systems under electrical load. A constant voltage of 9 V was applied, causing a current of 1 A to flow through the bonded system. The overlap of the two aluminum specimens bonded with the TQ-based adhesive defined a specific adhesive area where process heat was generated due to Joule heating.

To ensure reproducible thermographic imaging, the surfaces under investigation were pre-coated with LabIR paint to achieve high and uniform emissivity (Figure S36A). Thermographic acquisition started prior to the activation of the power supply to precisely document the initial thermal state.

For analysis, four measurement points were defined. One point centrally located above the adhesive area on the upper metal strip, one point near the adhesive area on the lower metal strip, and one point outside the adhesive region on each of the two metal strips.

Based on the recorded image sequences, temperature-time diagrams were generated, illustrating both the maximum temperature and the heating behavior at each measurement point. Different adhesive formulations were investigated, including a fully formulated adhesive containing carbon black (CB) and ionic liquid (IL), as well as a variant filled with CB only.

It was observed that the fully formulated adhesive exhibited only a moderate temperature increase up to approximately 26 °C (Figure 4C, maintext), whereas the CB-filled variant showed a significant temperature rise to 75 °C (Figure S36 B, C). The results indicate significant differences in thermal conductivity and heat generation among the respective formulations.

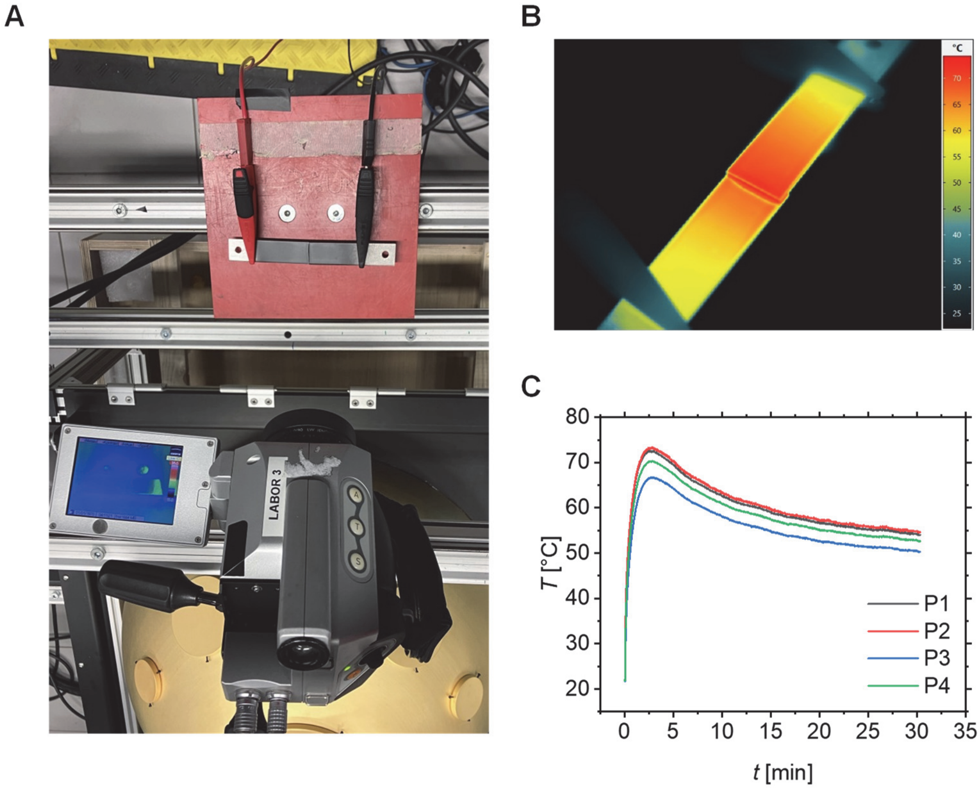

Figure S36. Experimental setup of the thermographic analysis (A). Thermographic image of the CB-filled TQ/ETTMP$_{700}$ adhesive between two aluminum specimens at maximum heat development (B). Tracking the temperature during the debonding process of the TQ/ETTMP$_{700}$/CB adhesive at various points (P1–P4; see Figure 4C in the main manuscript) on the specimens (C).

A decrease in current flow is observed in parallel with the drop in temperature (Fig. S35).

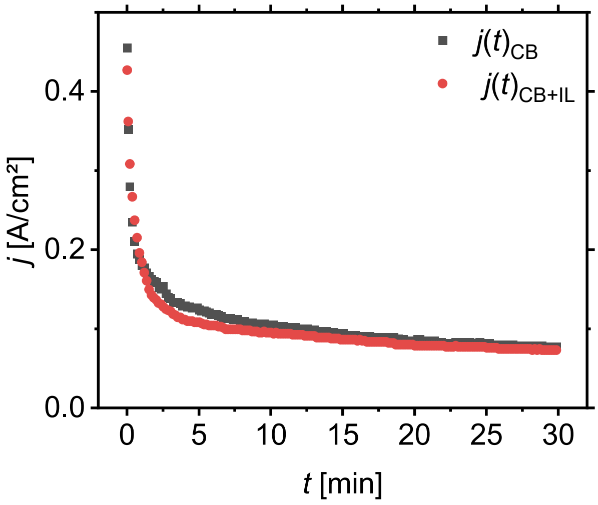

Figure S37. Current-time curve of the debonding processes at an applied constant voltage of 9 V.

S5.9 Cyclic voltammetry (CV)

S5.9.1 Two-electrode CVs

The CVs of the adhesive joints were recorded by connecting one aluminum substrate of the symmetrical Al/adhesive/Al samples to the working electrode and the other substrate to the counter and reference electrodes. The voltage was scanned between 0 and 15 V. Scanning below 0 V was avoided to prevent reversing the direction of the current, in order to clearly separate the anode and cathode.

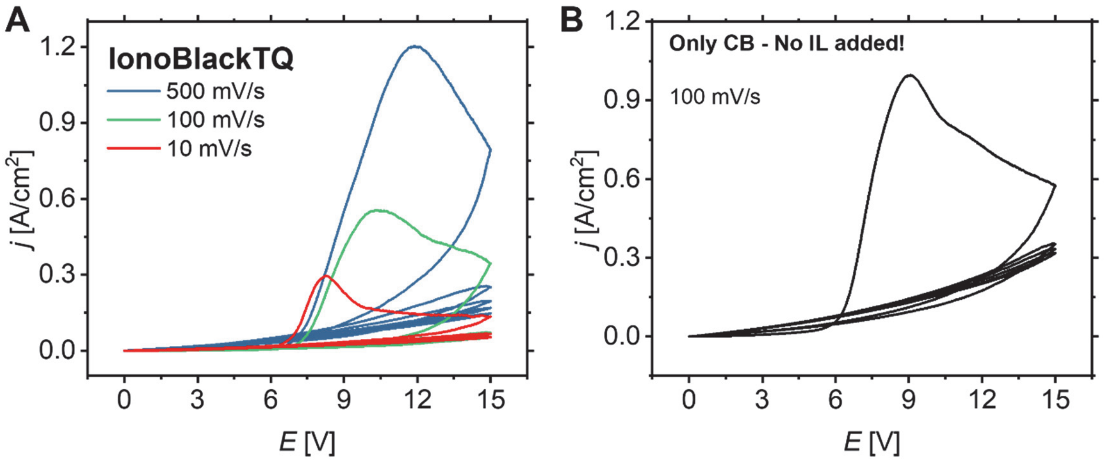

Figure S38. Two-electrode (2EL) CVs of IonoBlackTQ between two aluminum substrates at given scan rates (A) and of TQ/ETTMP$_{700}$/CB 100 mV·s^{-1} (B).

S5.9.2 Three-electrode CVs

CVs were recorded using a freshly polished gold electrode (1 mm diameter) as working electrode, a Pt wire as counter electrode and a silver wire as pseudo reference. All species were first measured in the electrolyte. Subsequently, ferrocene (Fc) was added and the measurement repeated. The potential was adjusted to the $E_{1/2}$ recorded for the Fc/Fc$^+$ redox couple as reference point.[12] Finally, the CVs without Fc were aligned with those containing Fc. For the blank electrolyte, the measurement containing Fc is shown as an example. Besides the Fc/Fc$^+$ redox couple, the DMF/Bu_4N·BF_4 electrolyte shows characteristic minor currents. The potential window of the electrolyte is limited to approx. -3 to +1 V vs Fc/Fc+.

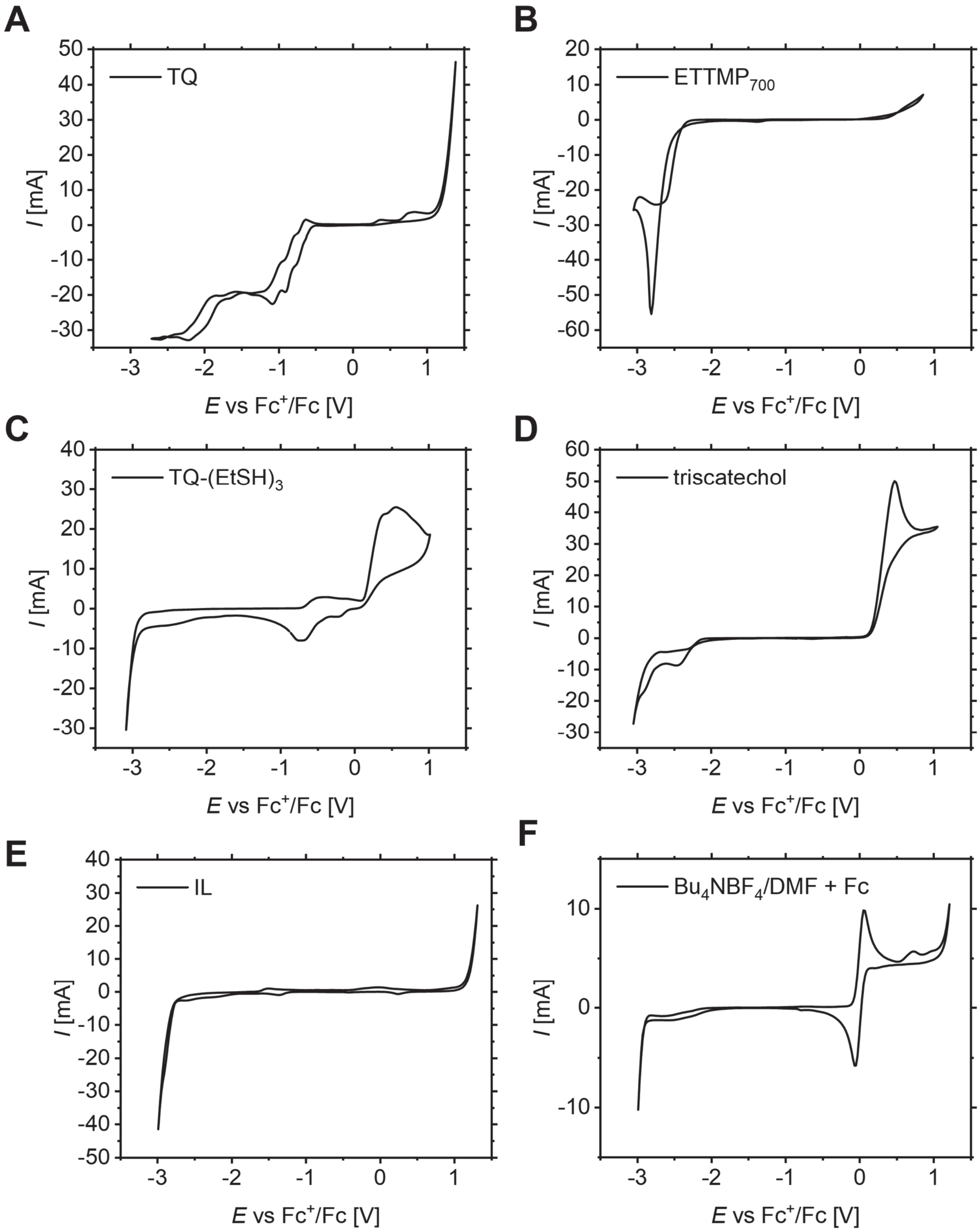

Figure S39. CVs in DMF/$Bu_4N{\cdot}BF_4$ with gold electrode as working electrode, a platinum wire as counter electrode and a silver wire as pseudo reference of TQ (A), $ETTMP_{700}$ (B), TQ-EtSH-TCC (C), triscatechol (D), BMIM·NTf_2 as ionic liquid (IL) (E) and a blank measurement with just the electrolyte and ferrocene (Fc) (F). The potential was adjusted to the $E_{1/2}$ recorded for the Fc^+/Fc redox couple as reference in all measurements, which is only shown for the blank electrolyte in F.

S5.10 Mini display model study

S5.10.1 Preliminary experiments

In preliminary experiments, it was first investigated whether non-conductive materials such as glass could be rendered electrically conductive through printing with silver ink. For this purpose, glass test specimens were printed with a silver ink, which was then dried at 120 °C for 60 min (Figure S38A). Subsequently IonoBlackTQ$_{1.1}$ was used to bond the glass specimen to an aluminum substrate in an overlapping configuration (Figure S40B). The adhesive was applied at only four discrete points to minimize the bonded area and avoid substrate failure (glass breakage).

The bonded assemblies, cured at 120 °C for 17 h, were then subjected to an electrical load of 9 V under a mechanical preload of 500 g. Within 5 min under these conditions, failure occurred with a complete adhesive failure between the adhesive and the aluminum specimen (Figure S38C). In contrast, the same bonded systems that were subjected only to the 500 g preload. Here without electrical stress, did not fail during the same period. These control samples only separated during subsequent mechanical lap shear testing reaching 1.3 ± 0.4 MPa and showing delamination of the silver ink (Figure S40D).

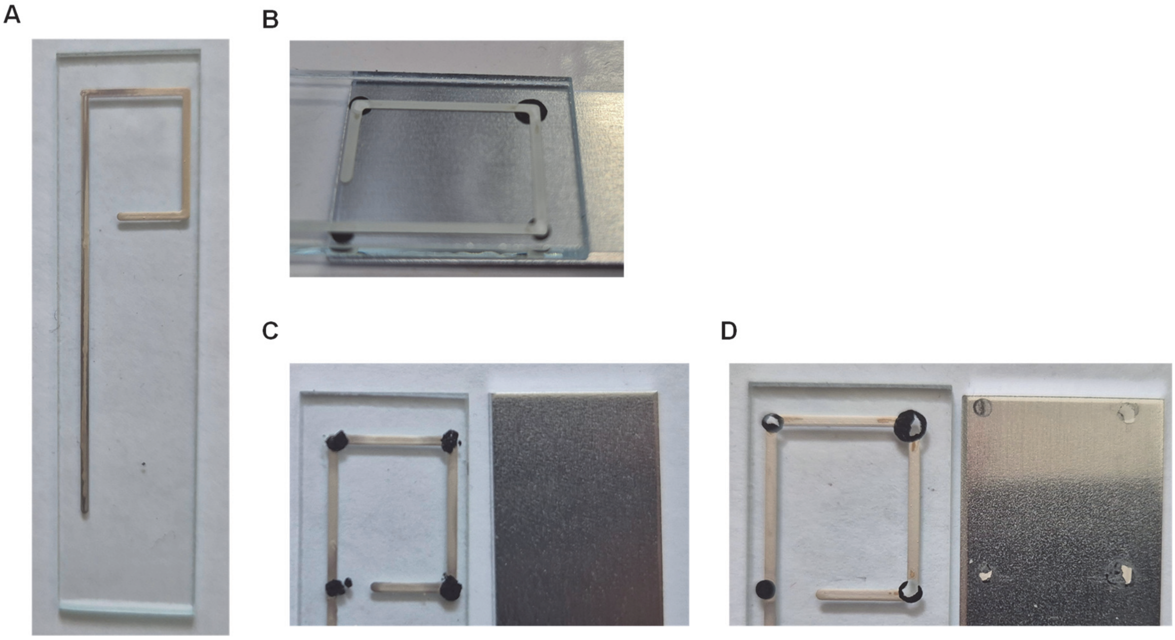

Figure S40. Preliminary experiments for electrochemical debonding on a mini display model. Cured silver ink printed on glass (A). Bonded (glass/Ag-ink/IonoBlackTQ1.1/Al) system (B). Tested system after voltage application (9 V, 5 min) (C) and after lab shear testing (D).

S5.10.2 Building the mini display model

To demonstrate the practical applicability of the adhesive using a model system, a 0.96" OLED display was equipped with a frame printed from Formlabs Grey Resin V4. A strip of conductive aluminum adhesive tape was applied to the display to facilitate easier connection with a clamp later on. Subsequently, silver ink was printed onto the surface using the V-ONE printer. The system was then allowed to dry at 90 °C for 2 days. While the manufacturer recommends drying at 120 °C, the extended drying time was chosen to compensate for the lower temperature.
After drying, four adhesive dots of the IonoBlackTQ$_{1.1}$ were manually applied to the silver tracks. The assembly was then cured again at 90 °C for 17 hours with the support of foldback clams.
The average diameter of the adhesive dots was measured using a caliper and found to be 4 ± 2 mm^2, resulting in a total bonded area of approximately 16 mm^2. This corresponds to a minimum bond strength of 0.3 MPa under a 500 g load.

S5.11 Investigation of fracture pattern surface morphology

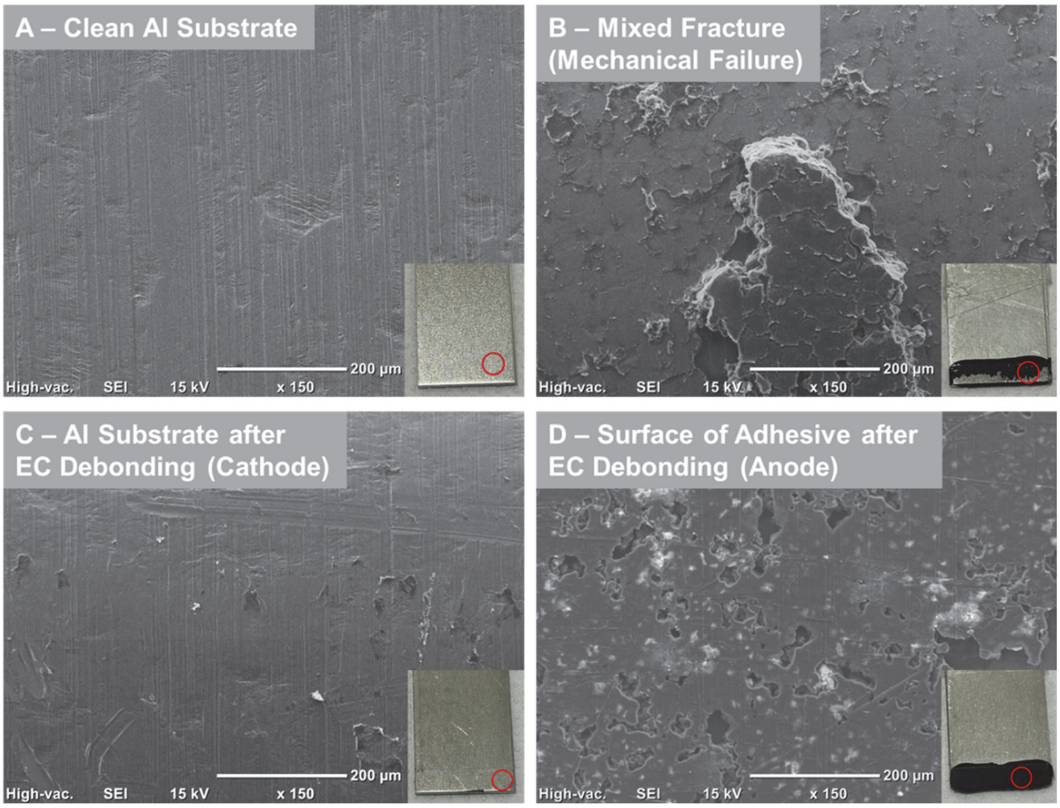

Figure S41. Surface morphology of aluminum substrates and IonoBlackTQ. Clean aluminum substrate without any adhesive (A). Failed adhesive after lap shear test with mixed fracture pattern (B). Electrochemically (EC) debonded substrate (Cathode, C) and adhesive (Anode, D) after separation of the adhesive joint. The insert shows the measured samples with the red circle indicating the area of origin of the SEM images.

The formulation of IonoBlackTQ allowed for the guided debonding of the adhesive with a purely adhesive fracture pattern after electrochemical debonding (Figure 4 in main text). Without the electrochemical debonding, the fracture pattern was mixed between adhesive and cohesive failure. SEM was used to investigate the surface morphology of the fractured adhesive joints (Figure S41). After lap shear testing without electrochemical treatment, a thin layer of the adhesive appears to remain on the aluminum substrate surface (Figure S41B). In that case, the typical pattern of the aluminum as seen in Figure S41A is not exposed indicating cohesive failure. In contrast, after electrochemical debonding the fracture pattern is almost fully adhesive. The majority of the adhesive is removed from the substrate, which appears almost identical in surface structure to the original aluminum surface, with only a few residuals remaining (Figure S41C). The debonded adhesive, on the other substrate side, exhibits a remarkably flat surface (former adhesive interface), partially even replicating the machining marks of the aluminum substrate (Figure S41D). This highlights the dramatic change in fracture behavior and the reduction of adhesive strength induced by electrochemical debonding of IonoBlackTQ.

S6 References

[1] M. Frigerio, M. Santagostino, S. Sputore, *J. Org. Chem.* **1999**, *64*, 4537-4538.
[2] J. M. Krüger, H. G. Börner, *Angew. Chem. Int. Ed.* **2021**, *60*, 6408-6413.
[3] J. B. H. Plumb, Donald Joe; , *Chemical & Engineering News Archive* **1990**, *68*, 2-3.
[4] E. H. Vickery, L. F. Pahler, E. J. Eisenbraun, *J. Org. Chem.* **1979**, *44*, 4444-4446.
[5] J. M. Krüger, C.-Y. Choi, F. Lossada, P. Wang, O. Löschke, D. Auhl, H. G. Börner, *Macromolecules* **2022**, *55*, 989-1002.
[6] J. Pei, C.-C. Hsu, Y. Wang, K. Yu, *RSC Adv.* **2017**, *7*, 43540-43545.
[7] a) W. M. A. Niessen, in *Ion/Molecule Attachment Reactions: Mass Spectrometry* (Ed.: T. Fujii), Springer US, Boston, MA, **2015**, pp. 319-328; b) C. S. Kaddis, S. H. Lomeli, S. Yin, B. Berhane, M. I. Apostol, V. A. Kickhoefer, L. H. Rome, J. A. Loo, *J. Am. Soc. Mass. Spectrom.* **2007**, *18*, 1206-1216; c) M. S. Lee, M. Zhu, *Mass spectrometry in drug metabolism and disposition: Basic principles and applications*, John Wiley & Sons, **2011**.
[8] Y. Zhang, Q. Zhao, H. Shao, S. Zhang, X. Han, *Advances in Materials Science and Engineering* **2014**, *2014*, 107375.
[9] R. Mahou, C. Wandrey, *Polymers* **2012**, *4*, 561-589.
[10] D. F. Duxbury, *Chem. Rev.* **1993**, *93*, 381-433.
[11] a) J. Yang, M. A. Cohen Stuart, M. Kamperman, *Chem. Soc. Rev.* **2014**, *43*, 8271-8298; b) H. Choi, K. Lee, *Appl. Sci.* **2022**, *12*, 11626.
[12] G. Gritzner, J. Kuta, *Pure Appl. Chem.* **1982**, *54*, 1527-1532.
